# Modulation of Growth, Intestinal Microbiota, and Immune Responses in *Cherax quadricarinatus* by Mannan Oligosaccharides

**DOI:** 10.3390/ani16162511

**Published:** 2026-08-12

**Authors:** Ke-Xiang Ding, Ze-Long Zhang, Yao-Peng Lu, Yan-Lei Cao, Si-Qi Zhang, Pei-Hua Zheng, Jun-Tao Li, Xiu-Xia Zhang, Si-Meng Wang, Zhong-Hai Liao, Rui Sun, Jian-An Xian

**Affiliations:** 1 College of Biology and Agriculture, Jiamusi University, Jiamusi 154007, China; lucas.ding98@gmail.com (K.-X.D.); zsqdyx88@163.com (S.-Q.Z.); 19837213619@163.com (S.-M.W.); 2 Hainan Provincial Key Laboratory for Functional Components Research and Utilization of Marine Bio-Resources, Institute of Tropical Biosciences and Biotechnology, Chinese Academy of Tropical Agricultural Sciences, Haikou 571101, China; zhangzelong@catasitbb.cn (Z.-L.Z.); luyaopeng@catasitbb.cn (Y.-P.L.); m799846567@gmail.com (Y.-L.C.); zhengpeihua@catasitbb.cn (P.-H.Z.); lijuntao@catasitbb.cn (J.-T.L.); zhangxiuxia@catasitbb.cn (X.-X.Z.); 3 Hainan Nanfan Administration Bureau, Sanya 572000, China; liao.1124@163.com

**Keywords:** *Cherax quadricarinatus*, mannan oligosaccharides (MOS), growth performance, immune function, intestinal microbiota

## Abstract

This study evaluated the effects of mannan oligosaccharides (MOSs) on *Cherax quadricarinatus* and explored the underlying mechanisms. Crayfish were randomly assigned to six dietary treatments containing 0 (M0), 0.2 (M1), 0.4 (M2), 0.6 (M3), 0.8 (M4), and 1.0 (M5) g·kg^−1^ MOSs for an 8-week feeding trial. The results demonstrated that MOS supplementation significantly improved growth performance, with weight gain rate and specific growth rate in the M3 group being higher than those in the control (*p* < 0.05). Survival rate did not differ significantly among dietary treatments, although the M2 group showed the highest numerical value. MOSs significantly reduced muscle lipid content (*p* < 0.05) without affecting moisture, protein, or ash levels. Microbiota analysis revealed an increase in Firmicutes and a decrease in Proteobacteria in the M3 group (*p* < 0.05). Non-specific immune parameters, including alkaline phosphatase, acid phosphatase, total antioxidant capacity, superoxide dismutase, and glutathione peroxidase, were significantly elevated in the M2 and M3 groups, while malondialdehyde was reduced (*p* < 0.05). Immune-related genes, including glutathione peroxidase (GSH-Px), superoxide dismutase (SOD), thioredoxin (Trx), lysozyme (LZM), and anti-lipopolysaccharide factor (ALF), were upregulated in MOS-fed crayfish (*p* < 0.05). Following *Aeromonas hydrophila* challenge, survival rates were significantly higher in all MOS-treated groups (*p* < 0.05). These results indicate that MOS supplementation enhances growth performance, modulates gut microbiota composition, and strengthens immune responses and disease resistance in *C. quadricarinatus*.

## Simple Summary

Mannan oligosaccharides are prebiotic feed additives that may improve health and disease resistance in cultured aquatic animals. The redclaw crayfish (*Cherax quadricarinatus*) is an economically valuable freshwater-cultured crustacean, while bacterial diseases frequently restrict its healthy farming. This study evaluated graded dietary levels of mannan oligosaccharides supplemented to diets for redclaw crayfish over an 8-week feeding trial. Moderate MOS supplementation improved growth performance, enhanced antioxidant capacity and non-specific immune responses, modulated the intestinal microbiota structure by enriching beneficial bacterial taxa, and increased host resistance against *Aeromonas hydrophila* infection. Excessive MOS addition did not produce further beneficial effects and even caused partial performance decline. These findings suggest that appropriate mannan oligosaccharides can be adopted as promising functional feed additives for redclaw crayfish practical farming.

## 1. Introduction

Over the past few decades, the aquaculture industry has undergone a shift from low-intensity, extensive systems to highly intensive farming models. While this intensification has boosted productivity, it has also introduced challenges such as compromised water quality and a heightened vulnerability of aquatic species to disease outbreaks [1,2]. To address these issues in earlier stages of aquaculture development, antibiotics were commonly utilized as a primary intervention strategy. The detection of antibiotic residues in aquaculture products and the rapid development of resistance among pathogenic bacteria have drawn global attention, prompting policymakers to implement stringent restrictions or outright bans on antibiotic use in aquaculture [3]. As promising alternatives, polysaccharide-based immunostimulants have attracted increasing attention due to their abundant availability, low cost, and ease of application [4]. These compounds have been successfully applied in the culture of fish, shrimp, and crabs, exhibiting notable effects in enhancing growth performance and immunity [5].

Mannan oligosaccharides (MOSs) are polysaccharides widely distributed in nature, commonly found in the cell walls of yeast and other organisms [6]. They are chemically stable, non-toxic, and water-soluble and exhibit several beneficial properties, including promoting animal growth, modulating immune function, enhancing the structure of the intestinal microbiota, and exerting anti-inflammatory and antioxidant effects [7]. As a result, MOSs are widely recognized as a commonly used prebiotic. MOSs have been reported to alleviate glucose metabolism disorders induced by high-carbohydrate diets, thereby helping to maintain liver health in juvenile *Oreochromis niloticus* [8]. Additionally, MOSs can improve the growth performance of *E. sinensis*, enhance antioxidant capacity and non-specific immunity, and increase both the length and width of intestinal villi [9]. In *Dicentrarchus labrax*, intestinal administration of MOSs has been shown to improve resistance to *Vibrio anguillarum* and increase survival rates [10]. Moreover, D-mannose has been found to inhibit *Salmonella typhimurium* adhesion to the intestinal epithelium of chickens in vitro, thereby reducing its colonization in the gut [11].

*Cherax quadricarinatus*, commonly known as the redclaw crayfish, is a freshwater crustacean native to parts of Australia and New Guinea [12]. This species has gained popularity in aquaculture due to its high growth rate, tolerance to diverse water conditions [13,14] and suitability for cultivation in relatively confined spaces. It can be cultured in various aquaculture systems, including ponds [15] and tanks [16,17].

Mannan oligosaccharides (MOSs) have been reported to enhance growth performance indicators such as specific growth rate (SGR), weight gain rate (WGR), and survival rate (SR) in a range of cultured aquatic organisms. Due to their affordability and accessibility, MOSs are increasingly recognized as a beneficial dietary supplement in aquaculture. Wang et al. [18] reported that dietary MOSs improved growth performance and digestive enzyme activities in *Litopenaeus vannamei*. Novriadi et al. [19] found that yeast-derived MOSs enhanced survival and immune responses against *V. parahaemolyticus* in *L. vannamei.* Similarly, Hoang et al. [20] demonstrated that MOSs improved growth and survival in pompano (*Trachinotus ovatus*), reduced intestinal *Vibrio* populations, and increased feed and nutrient utilization efficiency. Harikrishnan et al. [21] also showed that MOS supplementation enhanced the feed conversion ratio (FCR) and protein efficiency ratio (PER) in *Chanos chanos*. However, the dose–response effects and mechanisms of MOSs in C. quadricarinatus remain poorly understood. In the present study, MOSs were applied as a dietary supplement in the culture of *C. quadricarinatus* with the aim of investigating their potential immunostimulatory effects in crustaceans. This study seeks to provide valuable insights into how specific MOS variants may enhance the immune response of redclaw crayfish and support their application as functional feed additives in sustainable aquaculture.

## 2. Materials and Methods

### 2.1. Diet Preparation

The composition of the basal diet utilized in the experiment is detailed in Table 1. MOSs were supplemented at the expense of cellulose, and the inclusion levels of protein-, lipid-, and energy-providing ingredients were kept constant across all diets to maintain an isonitrogenous and isoenergetic formulation. Six experimental diets were prepared by supplementing the basal formulation with graded levels of MOSs at 0.0 g/kg (M0), 0.2 g/kg (M1), 0.4 g/kg (M2), 0.6 g/kg (M3), 0.8 g/kg (M4), and 1.0 g/kg (M5). The MOSs (purity ≥ 93%) were purchased from Beijing Solarbio Science & Technology Co., Ltd. (Beijing, China). The experimental diets were dehydrated to achieve a moisture level below 100 g/kg and subsequently preserved at −20 °C prior to feeding.

### 2.2. Experimental Design

The juvenile *C. quadricarinatus* used in this study were obtained from a commercial aquaculture farm located in Haikou, Hainan Province, China. Upon arrival, the crayfish were acclimated in 500 L cylindrical tanks equipped with a recirculating water system. During the one-week acclimation period, they were fed the basal diet. After 24 h of fasting, a total of 720 juvenile *C. quadricarinatus* with an initial mean body weight of 0.28 ± 0.01 g were randomly assigned to six dietary treatments: M0, M1, M2, M3, M4, and M5. Each treatment group contained 120 crayfish and was divided into three replicate tanks, with 40 crayfish per tank. Each replicate was reared independently in a separate culture tank equipped with 30 uniformly arranged and reinforced PVC pipes as shelters. Crayfish were hand-fed twice daily at 08:00 and 18:00, and the daily ration was set at approximately 8% of the estimated biomass in each replicate tank. Uneaten feed was collected, dried, and weighed to calculate the actual feed intake and feed conversion ratio. The feeding trial lasted for 8 weeks. Throughout the experimental period, water quality parameters were maintained within optimal ranges: temperature 23–26 °C, pH 7.6–7.8, total ammonia nitrogen < 0.05 mg/L, nitrite nitrogen < 0.01 mg/L, and dissolved oxygen (DO) > 5.0 mg/L.

### 2.3. Sample Collection

At the end of the 8-week feeding trial, samples were collected from *C. quadricarinatus*. The total number of individuals and their mean body weight in each treatment group were documented prior to sampling. To facilitate hemolymph collection, crayfish were first subjected to cold anesthesia by immersion in ice water for approximately 5 min. Hemolymph samples were then drawn using a sterile 2.5 mL syringe containing an anticoagulant buffer composed of NaCl (26.3 g/L), glucose (18.0 g/L), citric acid (6.3 g/L), sodium citrate (6.7 g/L), and EDTA-2Na (2.9 g/L). The collected hemolymph was immediately centrifuged at 3000 rpm for 10 min at 4 °C to separate cellular components. The supernatant was collected and stored at −80 °C for subsequent physiological and biochemical analyses. Each crayfish was then dissected to separate the cephalothorax and abdomen. Hepatopancreas tissues were collected for enzymatic activity analysis and immune-related gene expression. The whole intestine was aseptically dissected from each sampled crayfish, rather than collecting a specific intestinal segment, and was immediately placed into a sterile tube, frozen in liquid nitrogen, and stored at −80 °C for subsequent intestinal microbiota analysis. Muscle tissues were collected for proximate composition analysis. All tissue samples were snap-frozen in liquid nitrogen immediately after collection and then stored at −80 °C for further analysis.

### 2.4. Growth Performance

Growth performance indicators included survival rate (SR), weight gain rate (WGR), and specific growth rate (SGR), which were calculated using the following formulas:

Survival rate (SR, %) = 100 × (N_F_/N_I_)

Weight gain rate (WGR, %) = 100 × (W_F_ − W_I_)/W_I_

Specific growth rate (SGR, %/d) = 100 × (Ln WF − Ln WI)/T

where N_I_ = initial number of individuals, N_F_ = final number of individuals, W_I_ = initial body weight (g), W_F_ = final body weight (g), and T = feeding duration (days).

Feed conversion ratio (FCR) = FI/WG

where FI is the total dry feed intake per tank, calculated as the total dry feed offered minus the dry uneaten feed recovered, and WG is the wet biomass gain per tank.

### 2.5. Proximate Composition Analysis

The experiment determined the moisture content using the drying method at 105 °C, according to method 934.01, AOAC, 2006 [22]. After drying, the samples were ground and nitrated using concentrated sulfuric acid. The crude protein content was measured using an automatic Kjeldahl nitrogen analyzer (FOSS Kjeltec 8400, Höganäs, Sweden) based on method 984.13, AOAC, 2006 [23]. The crude fat content was determined using the Soxhlet extraction method, with petroleum ether as the solvent, following method 2003.05, AOAC, 2006 [24]. Crude fiber content was measured by acid–alkali digestion according to AOAC Official Method 962.09 (AOAC, 2006) [25]. Organic Matter (OM) was determined by subtracting ash content from total dry matter (method no. 942.05, AOAC 2006) [24]. Gross energy (GE) of the experimental diets was determined using an adiabatic oxygen bomb calorimeter (Parr Instruments, Moline, IL, USA) [26].

### 2.6. Enzymatic Activity Assays

The physiological and immunological parameters in the hemolymph and hepatopancreas of *C. quadricarinatus* were measured, including the activities of acid phosphatase (ACP), alkaline phosphatase (AKP), superoxide dismutase (SOD), catalase (CAT), and glutathione peroxidase (GSH-Px), total antioxidant capacity (T-AOC) and malondialdehyde (MDA) content. All parameters were assayed using commercial diagnostic kits (Nanjing Jiancheng Bioengineering Institute, Nanjing, China) following the manufacturer’s protocols.

### 2.7. Gene Expression Analysis

Total RNA was extracted from the hepatopancreas of *C. quadricarinatus* using the Animal Total RNA Isolation Kit (RE-03011; Foregene, Chengdu, China). The concentration and purity of RNA were assessed spectrophotometrically, and RNA integrity was verified via 1% agarose gel electrophoresis. First-strand cDNA was synthesized from total RNA using the PrimeScript™ RT Reagent Kit with gDNA Eraser (Takara, Dalian, China) following the manufacturer’s instructions. The resulting cDNA was stored at −20 °C until used in quantitative real-time PCR (qPCR) assays. The β-Actin gene was selected as the internal control for normalization, and specific primers for it and other immune-related genes (Table 2) were generated with Primer Premier v5.0 (Premier Biosoft International, Palo Alto, CA, USA). Primer sequences were designed by referencing both our in-house transcriptome database and relevant published studies [27]. Amplification efficiency for each primer pair was determined using standard curves generated from serially diluted pooled cDNA. The amplification efficiency was calculated from the slope of the standard curve using the following equation: E (%) = [10^(−1/slope) − 1] × 100. Therefore, the values reported in Table 2 are expressed as percentages. qPCR was conducted using a Stratagene Mx^3^005P Real-Time PCR System (Agilent Technologies, Agilent Technologies Inc., Santa Clara, CA, USA). Each qPCR reaction was performed in a 20 μL volume containing: 10.0 μL of 2× SYBR^®^ Green qPCR Master Mix (Takara Bio Inc., Kusatsu, Shiga, Japan), 1.0 μL of cDNA template, 0.4 μL each of forward and reverse primers (10 mM), and 8.2 μL of double-distilled water. The thermal cycling conditions were as follows: initial denaturation at 94 °C for 3 min, followed by 40 cycles of 94 °C for 15 s, 58 °C for 15 s, and 72 °C for 20 s. Relative gene expression levels were calculated using the 2^−ΔΔCt^ method.

### 2.8. Intestinal Microbiota Analysis

Genomic DNA was extracted from the intestinal samples using the HiPure Stool DNA Kit (Magen, Guangzhou, China). DNA quality was assessed by 1% agarose gel electrophoresis, and DNA purity was evaluated based on the A260/A280 ratio. The V3–V4 region of the bacterial 16S rDNA gene was amplified using the specific primers:

Forward: 5′-CCTACGGGNGGCWGCAG-3′;

Reverse: 5′-GGACTACHVGGGTATCTAAT-3′.

PCR products were purified, quantified, and used to construct sequencing libraries. Paired-end sequencing (PE250) was performed on the Illumina HiSeq 2500 platform (Illumina, Inc., San Diego, CA, USA). After sequencing, raw reads were filtered and merged into tags. Chimeric sequences were detected and removed, and the remaining high-quality tags were clustered into operational taxonomic units (OTUs) based on sequence similarity. Alpha diversity was assessed using coverage, ACE, Chao1, Shannon, and Simpson indices to evaluate within-sample diversity. Beta diversity was assessed using principal coordinate analysis (PCoA) based on Bray–Curtis dissimilarity to evaluate between-sample variation. LEfSe analysis was performed using the Kruskal–Wallis test followed by linear discriminant analysis. Taxa with an LDA score > 4.0 and *p* < 0.05 were considered significantly enriched.

### 2.9. Challenge Test

Aeromonas hydrophila used in the challenge experiment was isolated from diseased *C. quadricarinatus* and confirmed via biochemical identification. The strain was preserved in 25% glycerol at −80 °C until use. Prior to the experiment, the strain was activated and streaked onto LB agar plates and then incubated at 37 °C for 24 h. A single colony was transferred into 5 mL of LB broth and cultured in a shaking incubator at 180 rpm for 12 h at 37 °C. The bacterial concentration was determined using the plate counting method.

A preliminary experiment determined the 72-h median lethal dose (LD_50_) of *A. hydrophila* for *C. quadricarinatus* to be 1 × 10^8^ CFU/mL. For the challenge test, the activated bacteria were centrifuged at 6000 rpm for 2 min at 4 °C, resuspended in sterile phosphate-buffered saline (PBS), and adjusted to a final concentration of 1 × 10^8^ CFU/mL. At the end of the feeding trial, 15 crayfish were randomly selected from each replicate group for challenge. Each individual was injected intramuscularly with 15 μL of bacterial suspension into the third abdominal segment using a sterilized micro-syringe. Mortality was recorded every 12 h for 72 h. Dead crayfish were immediately removed and subjected to bacterial re-isolation to confirm infection with *A. hydrophila*.

### 2.10. Statistical Analysis

Experimental data were initially processed using Microsoft Excel and then analyzed using SPSS 18.0. Before statistical analysis, data were checked for normality using the Shapiro–Wilk test and for homogeneity of variance using Levene’s test. The tank was considered the experimental unit for all dietary treatment comparisons, and statistical analyses were performed using replicate tank means (*n* = 3 tanks per treatment). Data were analyzed by one-way analysis of variance (ANOVA), followed by Tukey’s multiple comparison test. Results are expressed as mean ± SD based on replicate tank means. Statistical significance was accepted at *p* < 0.05. Regression fitting analyses were conducted using Python 3.10 with relevant packages, including matplotlib, scipy, and sklearn. Alpha-diversity indices were compared among treatments using the non-parametric Kruskal–Wallis test because of the small number of biological replicates per group.

## 3. Results

### 3.1. Growth Performance

As shown in Table 3, the final body weight (FW), WGR, and SGR of crayfish in the M3 group were significantly higher than those in the M0 group (*p* < 0.05). No significant differences were observed between the M0 group and the other treatment groups (*p* > 0.05). Survival rates showed no significant differences between any groups (*p* > 0.05), although the M2 group exhibited the highest survival rate numerically.

**Figure 1:**
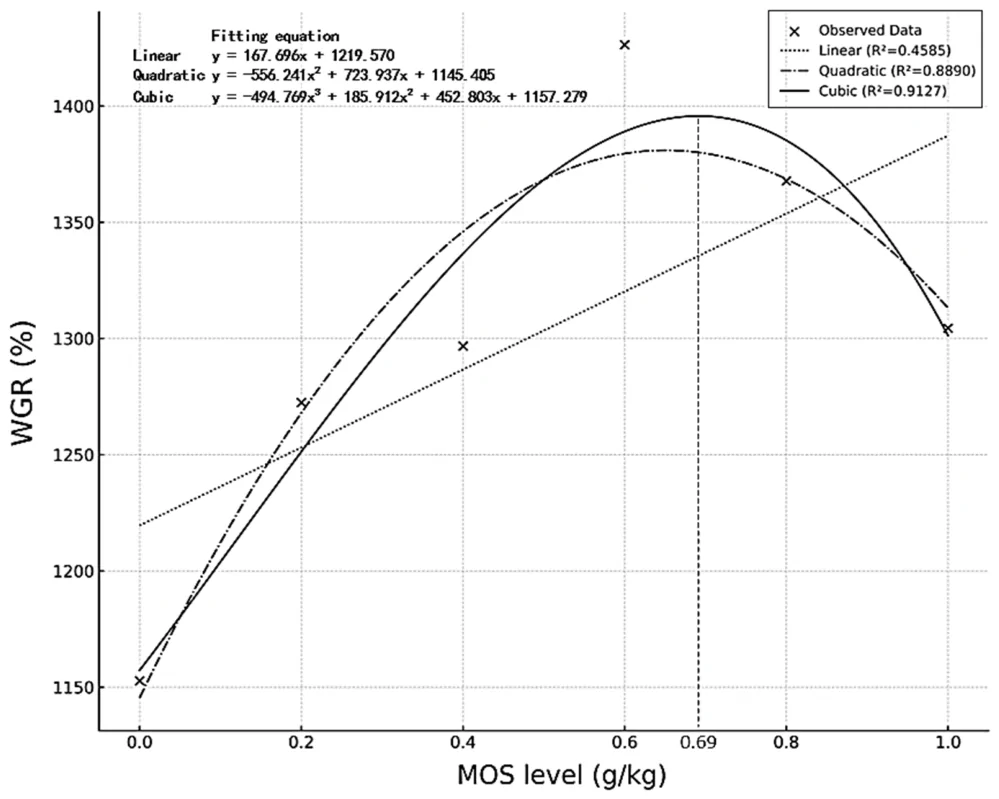
Regression curve depicting the relationship between dietary MOS inclusion level and WGR of *C. quadricarinatus*. Data were obtained after the 8-week feeding trial and are presented as means ± SDs based on three replicate tanks per treatment.

A regression analysis was performed with MOS supplementation levels (0–1.0 g/kg) as the independent variable and weight gain rate (WGR) as the dependent variable. The fitted curve is illustrated in Figure 1. Among the tested models, the cubic regression model provided the best fit, with the following equation: y = −494.769x^3^ + 185.912x^2^ + 452.803x + 1157.279, R^2^ = 0.9127. Based on this model, the predicted maximum WGR was achieved at an MOS supplementation level of 0.69 g/kg within the tested range (0–1.0 g/kg).

### 3.2. Muscle Composition

The effects of dietary MOS supplementation at different concentrations on the muscle composition of *C. quadricarinatus* are presented in Table 4. No significant differences (*p* > 0.05) were observed in moisture or crude protein contents among the treatment groups compared to the M0 group. However, crayfish in the M3 group exhibited the highest moisture and crude protein levels. Crude lipid content in muscle was significantly reduced (*p* < 0.05) in all MOS-supplemented groups compared to the control (M0), with the lowest value observed in the M3 group. No significant differences (*p* > 0.05) were detected in ash content among the groups.

**Figure 2:**
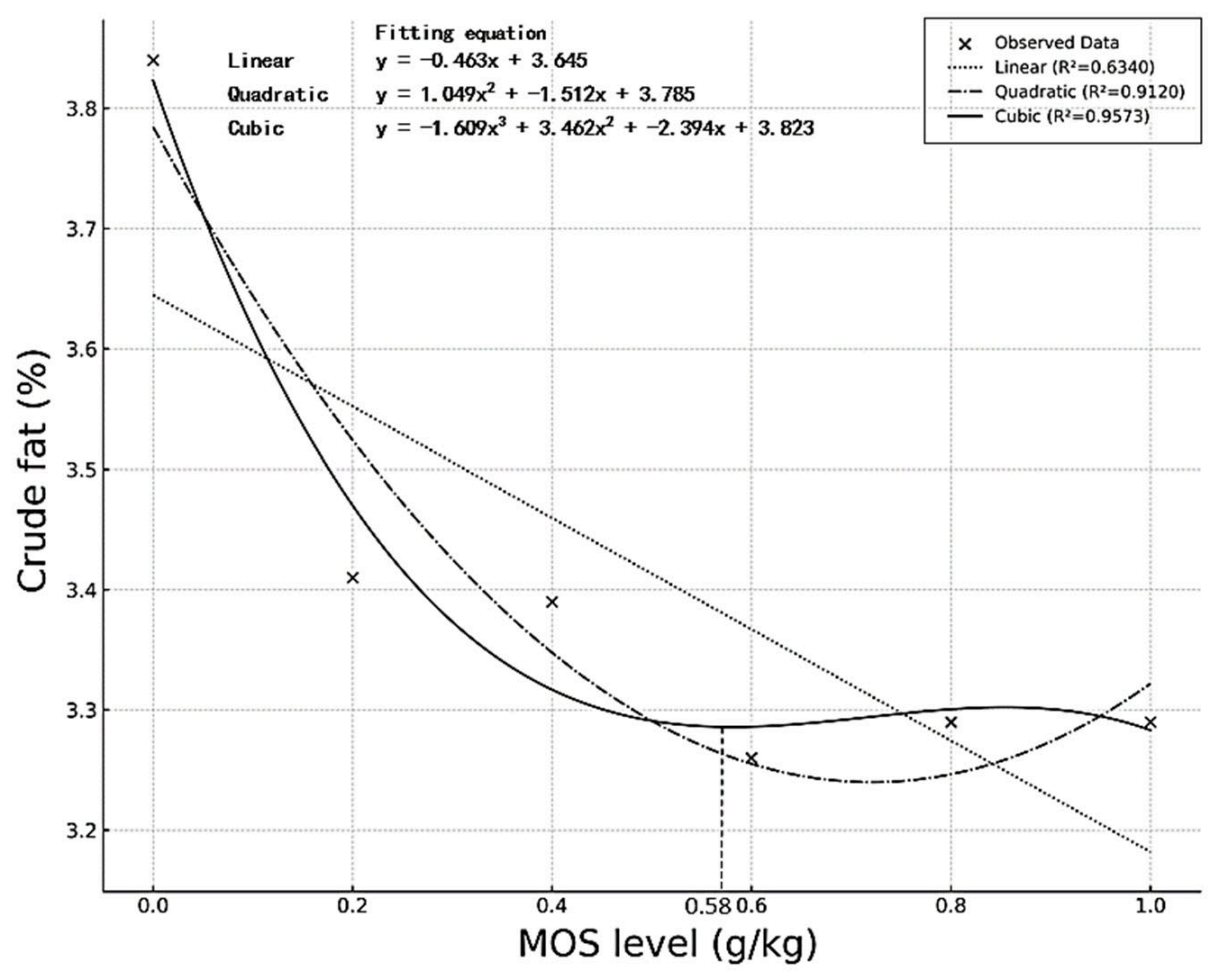
Fitted curve illustrating the relationship between dietary MOS supplementation level and crude lipid content in *C. quadricarinatus*. Data were obtained after the 8-week feeding trial and are presented as mean values based on three replicate tanks per treatment.

A regression analysis was conducted using MOS supplementation level (0–1.0 g/kg) as the independent variable and crude lipid content as the dependent variable. The fitted curve is illustrated in Figure 2.

Among the tested models, the cubic regression equation provided the best fit: y = −1.609x^3^ + 3.462x^2^ − 2.394x + 3.823, R^2^ = 0.9573. According to the model, the minimum predicted crude lipid content (3.29%) occurred at an MOS supplementation level of 0.58 g/kg within the tested range (0–1.0 g/kg).

### 3.3. Immune-Related Enzyme Activities

The effects of different dietary MOS supplementation levels on immune-related biochemical parameters in the hemolymph of C. quadricarinatus are summarized in Table 5. All experimental groups exhibited higher activities of immune-related enzymes compared to the control group (M0). Specifically, the activities of AKP, ACP, T-AOC, SOD, and GSH-Px were significantly increased (*p* < 0.05) in the M1, M2, and M3 groups. In the M4 group, significant increases (*p* < 0.05) were observed in AKP, ACP, SOD, and GSH-Px activities, while the M5 group showed significantly higher activities (*p* < 0.05) of AKP, ACP, and GSH-Px compared to M0. Moreover, the MDA content in hemolymph was significantly lower (*p* < 0.05) in all MOS-supplemented groups than in the control group.

The effects of graded dietary MOS supplementation on antioxidant and immune-related enzyme activities in the hepatopancreas of *C. quadricarinatus* are presented in Table 6. Compared with the control group (M0), all treatment groups showed enhanced antioxidant and immune capacities. In particular, the activities of AKP, ACP, SOD, CAT, GSH-Px, and T-AOC were significantly elevated (*p* < 0.05) in the M2 and M3 groups. Similarly, ACP, CAT, GSH-Px, and T-AOC activities in the M4 and M5 groups were significantly higher than those in the control group (*p* < 0.05), whereas AKP activity was significantly increased only in the M4 group. Additionally, ACP, GSH-Px, and T-AOC activities were significantly increased in the M1 group compared with M0 (*p* < 0.05). The MDA content in the hepatopancreas was significantly reduced (*p* < 0.05) in all MOS-supplemented groups.

**Figure 3:**
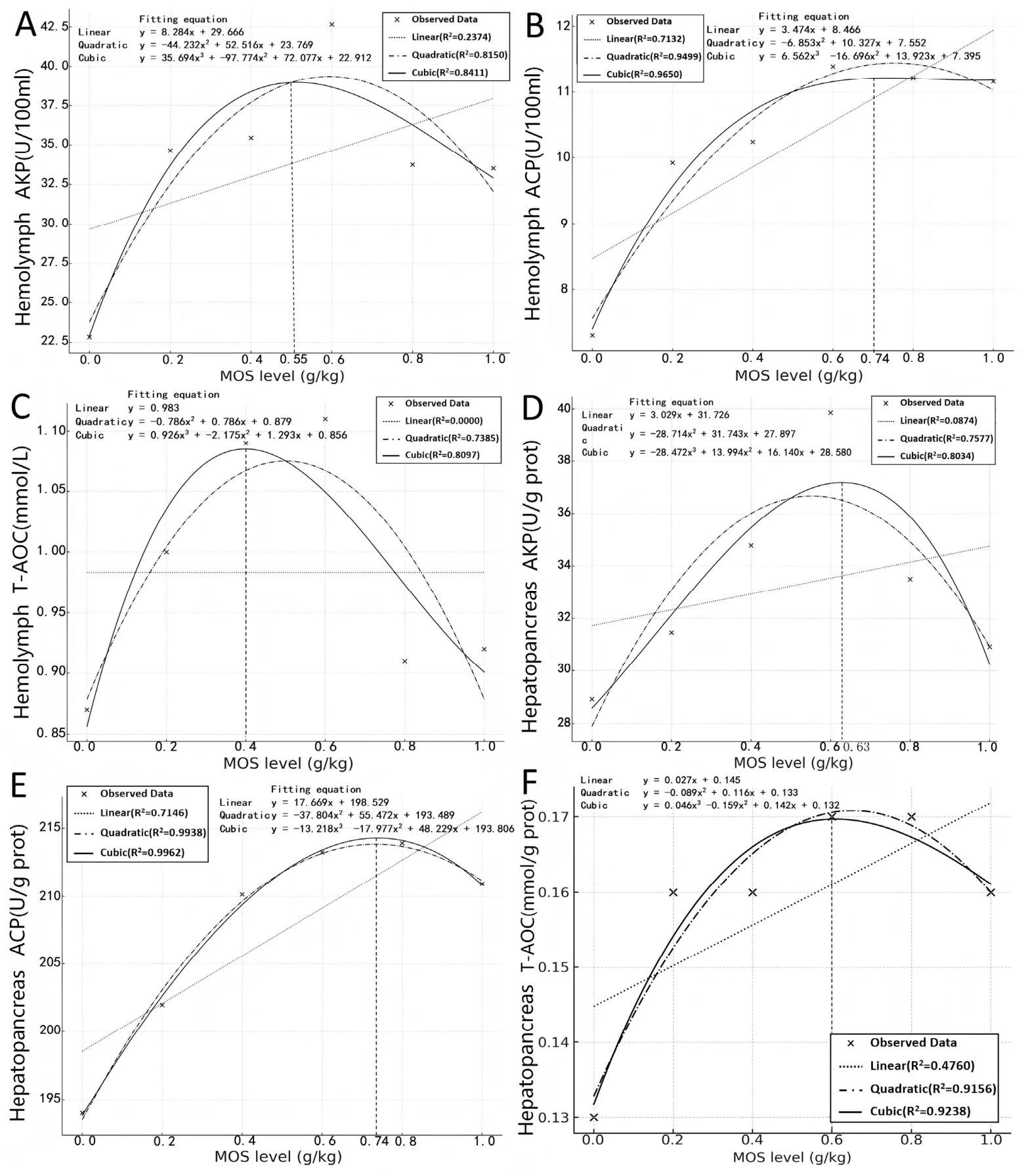
Regression curves showing the relationships between dietary MOS inclusion levels and immune- and antioxidant-related parameters in *C. quadricarinatus*. Data were obtained after the 8-week feeding trial and are presented as mean values based on three replicate tanks per treatment. (**A**) Hemolymph AKP activity; (**B**) Hemolymph ACP activity; (**C**) Hemolymph T-AOC content; (**D**) Hepatopancreas AKP activity; (**E**) Hepatopancreas ACP activity; (**F**) Hepatopancreas T-AOC content.

AKP and ACP activities were selected as indicators of immune capacity, while T-AOC was used as a marker for antioxidant status in *C. quadricarinatus*. Regression analyses were conducted with dietary MOS supplementation levels (0–1.0 g/kg) as the independent variable and AKP, ACP, and T-AOC values as dependent variables. The fitted curves are shown in Figure 3.

In hemolymph:

For AKP, the cubic regression equation was y = 35.694x^3^ + −97.774x^2^ + 72.077x + 22.912, R^2^ = 0.8411. The maximum predicted AKP activity (38.98 U/100 mL) was obtained at an MOS level of 0.55 g/kg.

For ACP, the best-fit cubic model was y = 6.562x^3^ − 16.696x^2^ + 13.923x + 7.395, R^2^ = 0.965. The maximum predicted ACP activity (11.21 U/100 mL) was reached at 0.74 g/kg.

For T-AOC, the cubic regression equation was y = 0.926x^3^ + −2.175x^2^ + 1.293x + 0.856, R^2^ = 0.8097. The maximum predicted T-AOC (1.08 mmol/L) was achieved at 0.40 g/kg MOS supplementation.

In hepatopancreas:

The cubic regression model for AKP was y = −28.472x^3^ + 13.994x^2^ + 16.140x + 28.580, R^2^ = 0.8034. The peak AKP activity (37.18 U/100 mL) was predicted at 0.63 g/kg MOSs.

For ACP, the equation was y = −13.218x^3^ − 17.977x^2^ + 48.229x + 193.806, R^2^ = 0.9962. The maximum predicted ACP activity (214.29 U/100 mL) occurred at 0.74 g/kg.

The T-AOC regression model was y = 0.046x^3^ − 0.159x^2^ + 0.142x + 0.132, R^2^ = 0.9238. The highest T-AOC value (0.17 mmol/g prot) was predicted at 0.60 g/kg MOSs.

### 3.4. Expression of Immune-Related Genes

**Figure 4:**
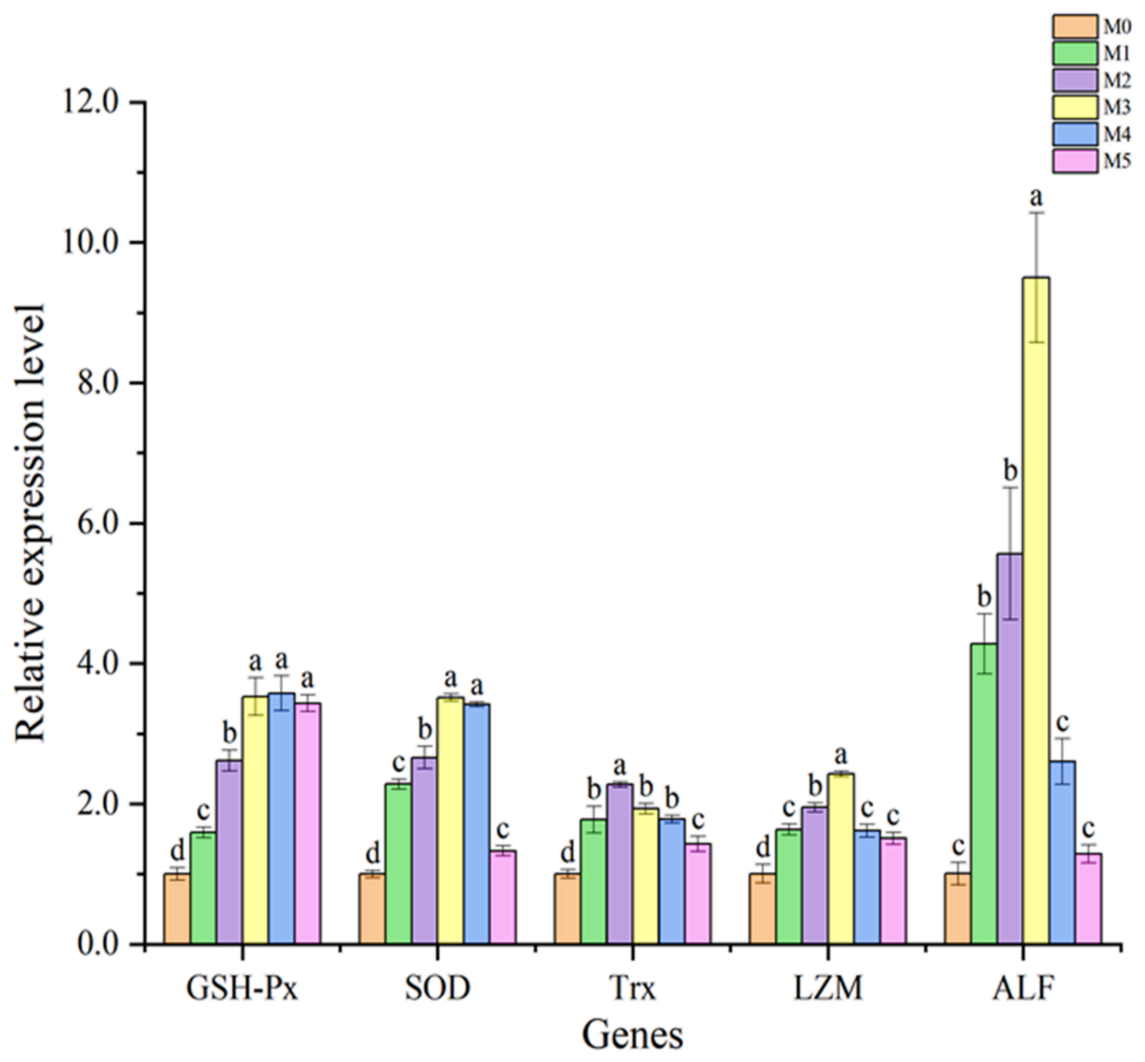
Effects of dietary MOS supplementation on the relative expression of immune-related genes in *C. quadricarinatus*. Samples were collected after the 8-week feeding trial. Data are presented as means ± SDs based on three replicate tanks per treatment. Different letters indicate significant differences among treatments (*p* < 0.05).

The expression levels of immune-related genes in the hepatopancreas of *C. quadricarinatus* are shown in Figure 4. All treatment groups exhibited upregulated gene expression compared to the control group (M0). Specifically, the expression levels of GSH-Px, SOD, Trx, LZM, and ALF were significantly upregulated (*p* < 0.05) in the M1, M2, and M3 groups. In the M4 and M5 groups, GSH-Px, SOD, Trx, and LZM levels were also significantly higher than those in the control (*p* < 0.05). Among all groups, the M3 group showed the highest expression levels of SOD, LZM, and ALF.

### 3.5. Intestinal Microbiota Analysis

The V3–V4 region of the 16S rDNA from 18 intestinal samples of *C. quadricarinatus* (with three biological replicates per treatment group, labeled as a, b, and c) was sequenced. A total of 2,786,564 paired-end reads were obtained. After quality control and merging, 2,779,914 clean reads were generated, with a minimum of 105,576 and an average of 154,440 clean reads per sample.

All sequences were clustered into operational taxonomic units (OTUs) at 97.0% similarity using USEARCH (v12) software. Detailed sequencing statistics for each sample are summarized in Table S1.

**Figure 5:**
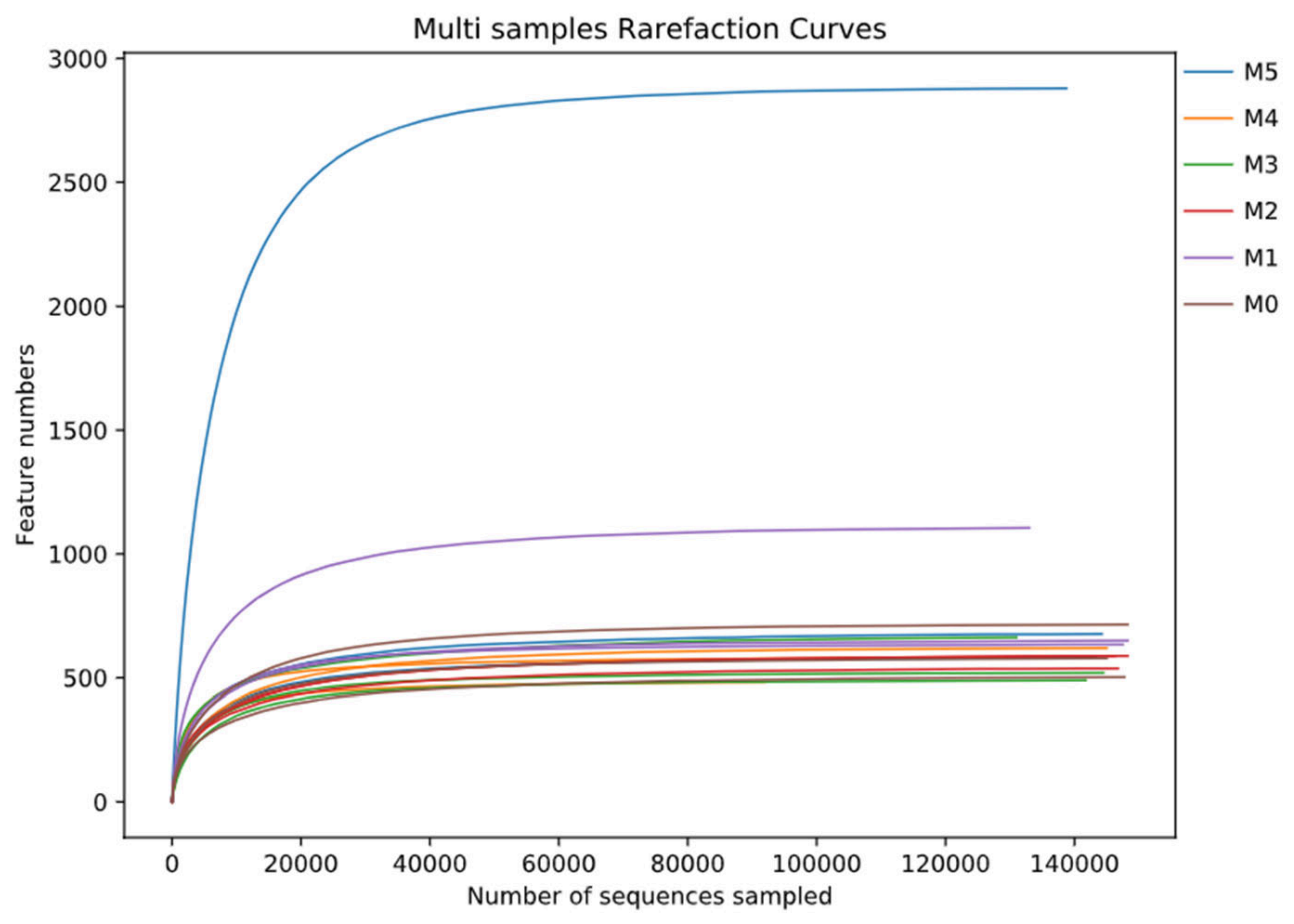
Rarefaction curves of intestinal microbiota samples from *C. quadricarinatus* under different dietary MOS treatments. Each curve represents one biological replicate. The rarefaction curves approach a plateau, and Good’s coverage values reach 100% in all groups, indicating that the sequencing depth was sufficient for downstream microbial diversity analysis.

Rarefaction curves are commonly used to assess whether sequencing depth is sufficient for downstream microbial diversity analysis. A steeply rising curve indicates insufficient sequencing, suggesting that more reads are needed; in contrast, a plateau suggests that the sequencing depth is adequate. As shown in Figure 5, all rarefaction curves tended to plateau when sequencing depth reached approximately 100,000 reads, indicating that the sequencing effort was sufficient and reliable for further analysis.

Alpha-diversity metrics, including Good’s coverage, ACE index, Chao1 index, Simpson index, and Shannon index, are summarized in Table 7. Good’s coverage reflects the probability of detecting species present in the sample. A higher value indicates a lower likelihood of undetected species. In this study, all groups exhibited 100% coverage, indicating that the sequencing depth was sufficient and species detection was complete, ensuring the reliability of the results. The ACE and Chao1 indices are indicators of species richness. No significant differences (*p* > 0.05) were found between the treatment groups and the control (M0), although the M1 group exhibited the highest ACE and Chao1 values, suggesting the greatest species richness. The Simpson and Shannon indices reflect species diversity. No significant differences (*p* > 0.05) were observed across groups; however, the M1 and M5 groups showed the highest Simpson index values, while the M5 group had the highest Shannon index, indicating that these two groups exhibited the greatest microbial diversity.

**Figure 6:**
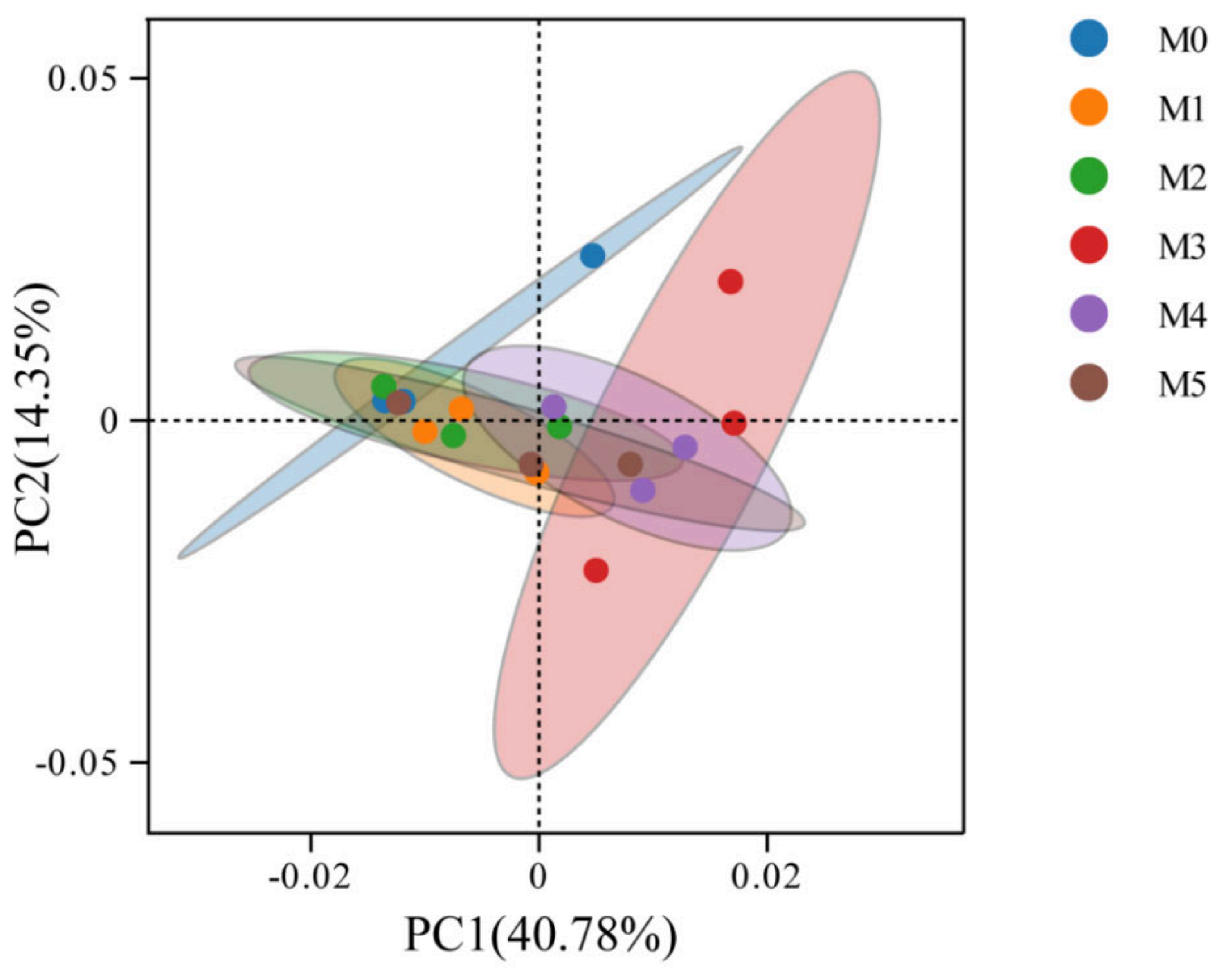
PCoA analysis of intestinal microorganisms of *C. quadricarinatus* based on Bray–Curtis dissimilarity. Samples were collected after the 8-week feeding trial, with three biological replicates per treatment.

As shown in Figure 6, principal coordinate analysis (PCoA) was conducted to assess the similarities and differences in intestinal microbial communities among samples from different treatment groups. The PCoA plot based on Bray–Curtis dissimilarity showed a treatment-associated clustering pattern among intestinal microbiota samples. PERMANOVA further confirmed that dietary MOS supplementation significantly affected the overall intestinal microbial community structure of *C. quadricarinatus* (R^2^ = 0.21, *p* = 0.002, Bray–Curtis, 999 permutations). These results indicate that MOSs altered the intestinal microbiota composition.

**Figure 7:**
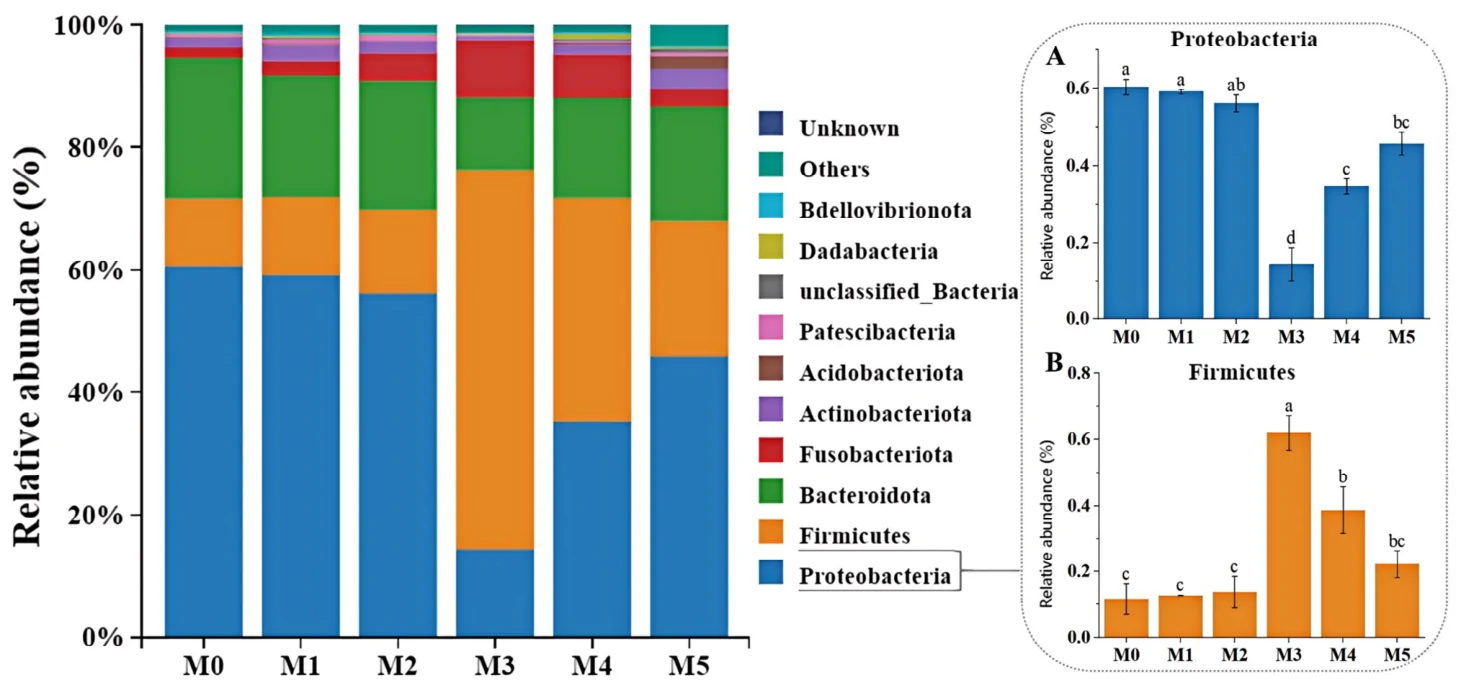
Phylum-level relative abundance of intestinal microbiota in *C. quadricarinatus*. Samples were collected after the 8-week feeding trial, with three biological replicates per treatment. (**A**) Relative abundance of Proteobacteria across different treatments; (**B**) Relative abundance of Firmicutes across different treatments. Values in the same row with the same lowercase letter are not significantly different (*p* > 0.05), based on one-way ANOVA and Duncan’s multiple-range test.

**Figure 8:**
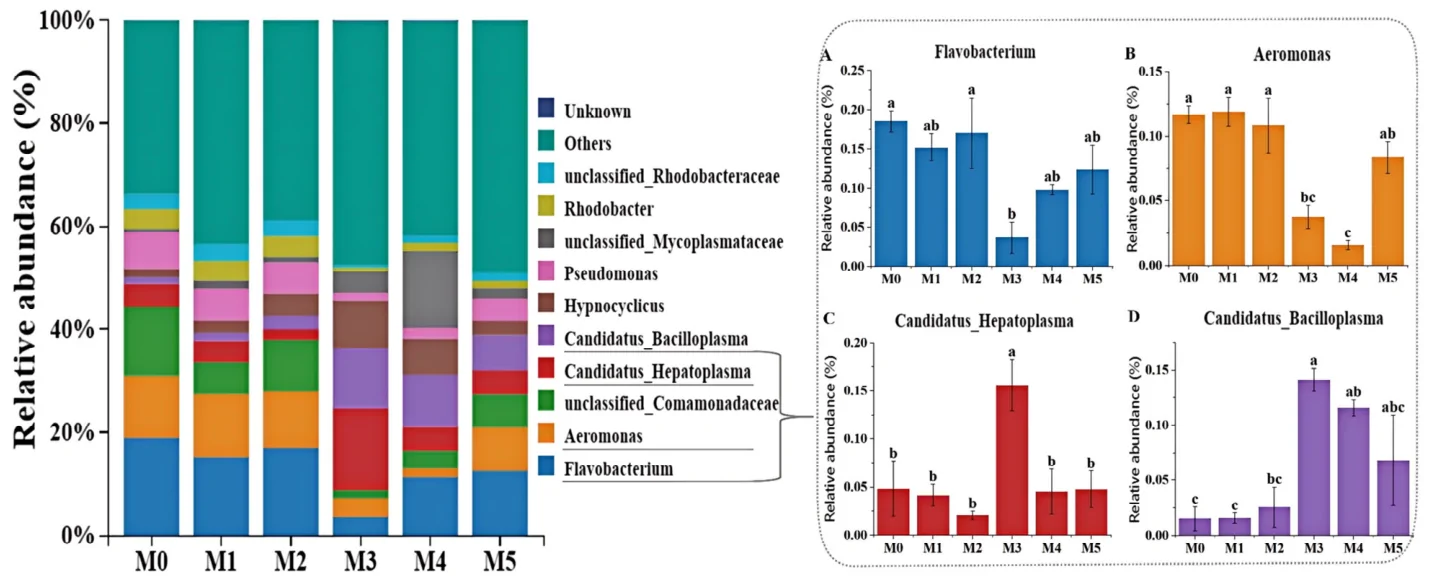
Genus-level relative abundances of intestinal microbiota in *C. quadricarinatus*. Samples were collected after the 8-week feeding trial, with three biological replicates per treatment. (**A**) *Flavobacterium*; (**B**) *Aeromonas*; (**C**) Candidatus_*Hepatoplasma*; (**D**) Candidatus_*Bacilloplasma.* Values in the same row with the same lowercase letter are not significantly different (*p* > 0.05), based on one-way ANOVA and Duncan’s multiple-range test.

**Figure 9:**
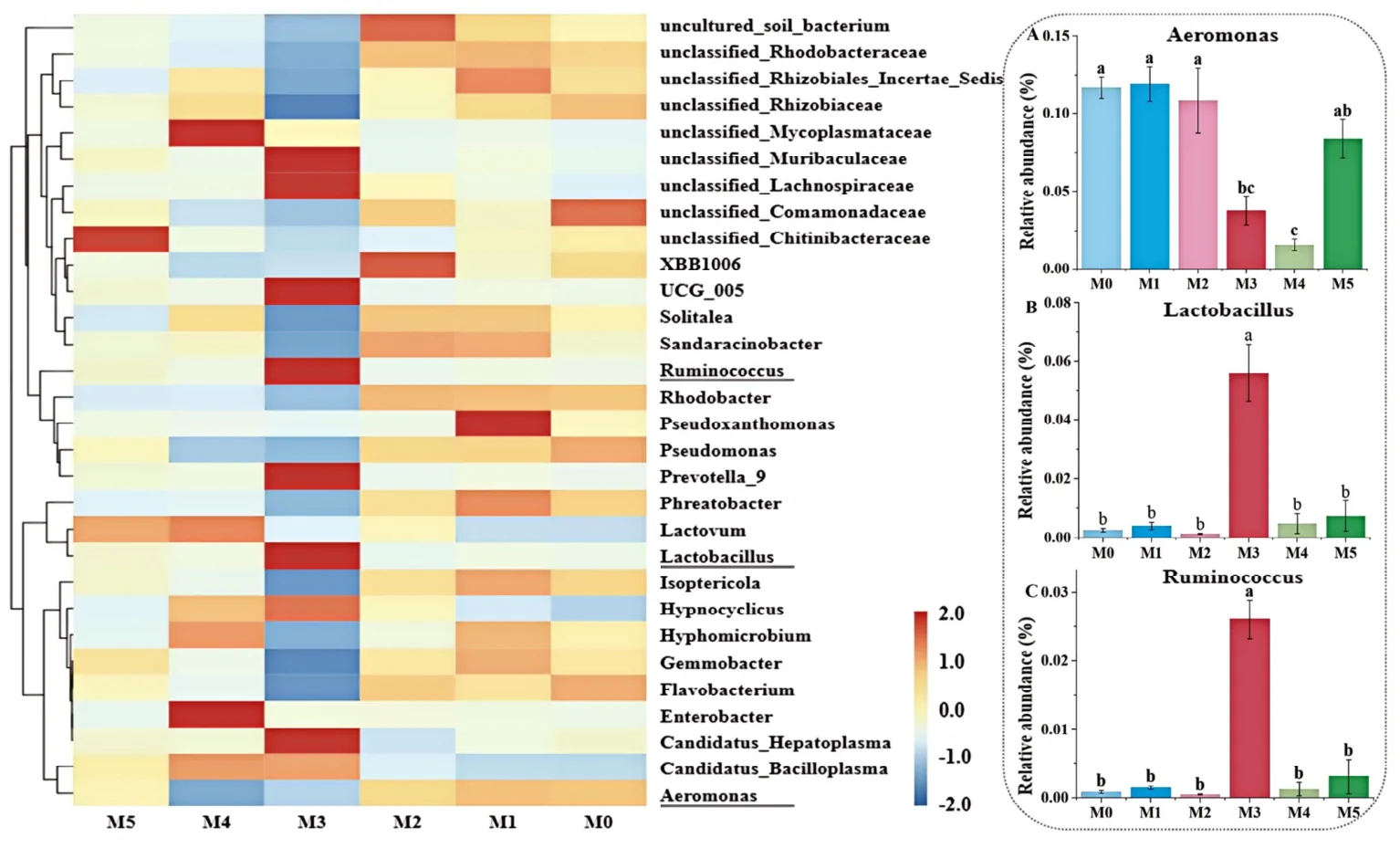
Heatmap showing the genus-level relative abundances of selected intestinal microbial taxa in *C. quadricarinatus*. Samples were collected after the 8-week feeding trial, with three biological replicates per treatment. (**A**) *Aeromonas*; (**B**) *Lactobacillus*; (**C**) *Ruminococcus.* Values in the same row with the same lowercase letter are not significantly different (*p* > 0.05), based on one-way ANOVA and Duncan’s multiple-range test.

The effects of dietary MOSs on the intestinal microbiota of *C. quadricarinatus* were analyzed at both the phylum and genus levels. At the phylum level (Figure 7), the top 10 most abundant phyla were visualized using stacked bar plots. The intestinal microbiota was primarily composed of Proteobacteria, Firmicutes, Bacteroidetes, and Fusobacteria. Dietary MOS supplementation influenced the relative abundances of these dominant phyla. Specifically, Proteobacteria showed a decreasing-then-increasing trend with increasing MOS levels, while Firmicutes exhibited the opposite pattern. In the M3 group, the relative abundance of Firmicutes was significantly increased, whereas Proteobacteria was significantly reduced compared to the control group (*p* < 0.05). At the genus level (Figure 8), the relative abundances of *Flavobacterium* and *Aeromonas* were significantly lower in the M3 group than in the M0 group (*p* < 0.05). In contrast, the abundances of Candidatus_*Hepatoplasma* and Candidatus_*Bacilloplasma* were significantly higher in the M3 group (*p* < 0.05). A genus-level heatmap (Figure 9) further confirmed that the abundance of *Aeromonas* was significantly reduced in the M3 group (*p* < 0.05), while the abundances of *Lactobacillus* and *Ruminococcus* were significantly increased (*p* < 0.05), indicating that MOS supplementation may enhance beneficial microbial populations while suppressing potential pathogens.

**Figure 10:**
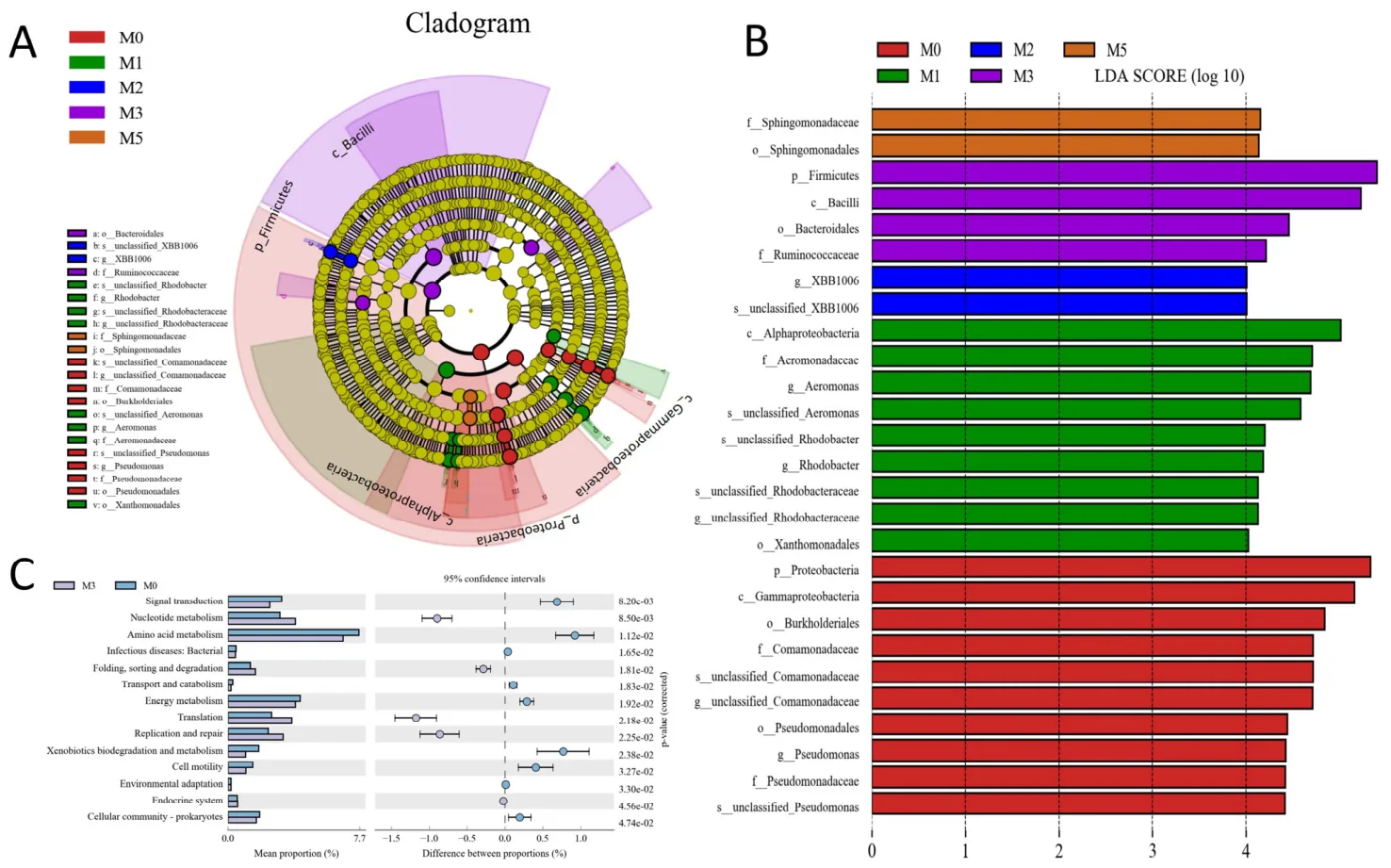
LEfSe and functional prediction analyses of intestinal microbiota in *C. quadricarinatus*. Samples were collected after the 8-week feeding trial, with three biological replicates per treatment. Taxa with an LDA score > 4.0 and *p* < 0.05 were considered significantly enriched. (**A**) LEfSe analysis cladogram; (**B**) LDA value distribution bar graph; (**C**) comparative analysis of KEGG metabolic pathway enrichment in the intestinal microbiota of *C. quadricarinatus* between M0 and M3 groups.

As shown in Figure 10A,B, LEfSe analysis identified 10 biomarkers in the M0 group, nine in M1, two in M2, four in M3, 0 in M4 and two in M5, respectively. Based on PICRUSt2 functional prediction results comparing the M0 and M3 groups, amino acid metabolism was the most frequently predicted microbial function. Furthermore, KEGG pathway enrichment analysis revealed that the top three predicted pathways with the highest microbial gene abundances were amino acid metabolism, energy metabolism, and nucleotide metabolism. Among them, nucleotide metabolism was significantly enriched in the M3 group compared to the M0 group (*p* < 0.05; Figure 10C).

### 3.6. Challenge Test

**Figure 11:**
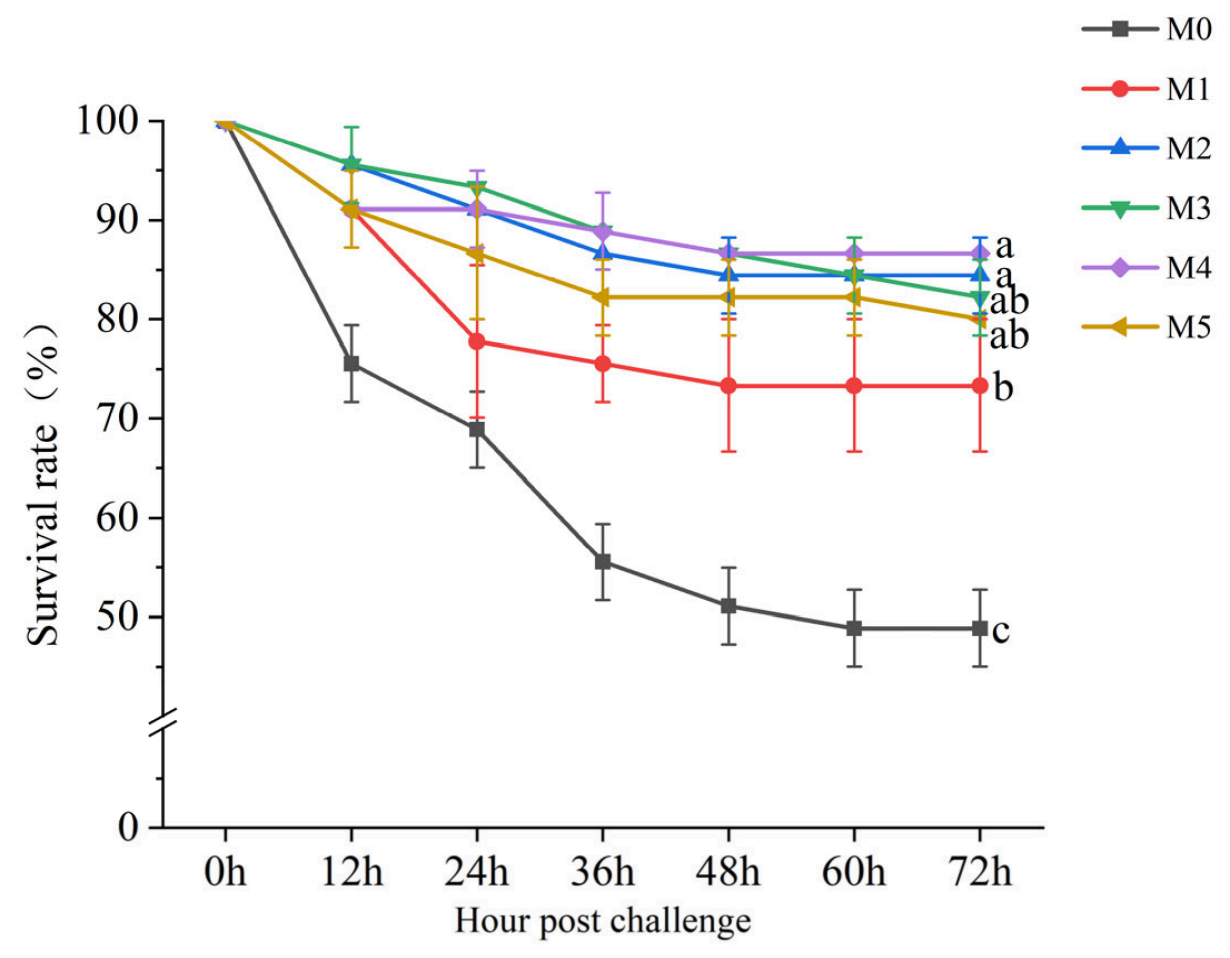
Effects of MOSs on the resistance of *C. quadricarinatus* to *Aeromonas hydrophila.* The challenge test lasted 72 h after *A. hydrophila* injection, with three replicate tanks per treatment. Values in the same row with the same lowercase letter are not significantly different (*p* > 0.05), based on one-way ANOVA and Duncan’s multiple-range test.

The effects of dietary MOS supplementation on the resistance of *C. quadricarinatus* to *A. hydrophila* infection are shown in Figure 11. After 72 h of challenge with *A. hydrophila*, the survival rates of *C. quadricarinatus* in the different groups were as follows: M0 group: 48.89 ± 3.85%; M1 group: 73.33 ± 6.67%; M2 group: 84.44 ± 3.85%; M3 group: 82.22 ± 3.85%; M4 group: 86.67 ± 0.00%; M5 group: 80.00 ± 0.00%. The survival rates of all MOS-treated groups were significantly higher than that of the control group (*p* < 0.05), indicating enhanced resistance to pathogenic infection. The highest survival rate was observed in the M4 group.

## 4. Discussion

The results of this study demonstrated that dietary supplementation with MOSs positively influenced the FBW, WGR, and SGR of *C. quadricarinatus*, with the predicted maximum WGR occurring at 0.69 g/kg based on cubic regression analysis. However, the responses observed at the highest MOS level (M5, 1.0 g/kg) suggest a slight leveling-off effect, as several enzyme activities and immune-related gene responses did not further increase compared with the intermediate-MOS groups. These findings align with previous studies across various aquaculture species. For example, Wang et al. [28] reported that MOS supplementation enhanced both weight gain and body length in juvenile *Oreochromis niloticus*. Similarly, Zhang et al. [29] observed improvements in WGR and SGR in *L. vannamei* fed MOS-enriched diets. In *Clarias batrachus*, Akter et al. [30] found that MOSs significantly improved growth performance. Positive effects of MOSs on growth have also been reported in *Ctenopharyngodon idella* [31], *Sparus aurata* [32], and *Paramisgurnus dabryanus* [33], supporting the outcomes of the present study.

The existing literature suggests that the mechanisms by which MOSs improve growth performance in aquatic animals include: ① enhancement of intestinal morphology: MOSs increase the length and width of intestinal villi, thereby expanding the surface area available for nutrient absorption and improving digestive efficiency [34,35]; ② modulation of gut microbiota composition: MOSs promote the proliferation of beneficial bacteria while suppressing harmful microbes, reducing nutrient loss to pathogenic competition and enhancing host nutrient availability [36]; ③ stimulation of digestive enzyme activity: MOS-containing diets have been shown to enhance the activity of digestive enzymes, further facilitating nutrient breakdown and absorption, thus contributing to improved growth performance [37].

Muscle moisture, crude protein, crude lipid, and ash content are considered key indicators for evaluating muscle quality in aquatic animals. Moisture content influences texture and mouthfeel; crude protein reflects the nutritional value; crude lipid is associated with meat tenderness and flavor; and ash represents the inorganic mineral and salt content remaining after oxidation. The present study revealed that dietary MOS supplementation significantly reduced the crude lipid content in the muscle of *C. quadricarinatus*, with the lowest lipid level observed at an inclusion rate of 0.58 g/kg. This reduction may be related to MOS-mediated modulation of gut microbiota and lipid metabolism. Interactions between the gut microbiota and lipid metabolic processes have been reported in aquatic organisms [38]. However, because intestinal lipid absorption and lipid excretion were not directly assessed in this study, further investigation is required to clarify the underlying mechanism. Similarly, Hoang et al. [20] reported that MOS supplementation increased muscle protein content while decreasing lipid content in pompano (*Trachinotus ovatus*). This may be related to the potential effects of MOS-containing prebiotic diets on body composition and lipid metabolism; however, the cited study used MOSs in combination with inulin, so the effect cannot be attributed solely to MOSs [39].

Unlike vertebrates, invertebrates lack an adaptive immune system and rely solely on innate immune defenses, including the prophenoloxidase (proPO) activation system, endogenous antimicrobial peptides, and phagocytosis [40,41]. As a result, the antioxidant defense system plays an even more critical role in invertebrates than in vertebrates. Immune- and antioxidant-related parameters such as ACP, AKP, and T-AOC are essential in preventing cellular damage caused by the accumulation of reactive oxygen species (ROS) generated during immune responses. Innate immunity serves as the sole defense mechanism for crustaceans against pathogenic microorganisms, and non-specific immune enzymes and antioxidant enzymes are central to this process. The present study showed that dietary MOS supplementation significantly increased the activities of AKP, ACP, SOD, CAT, and GSH-Px, as well as T-AOC levels, while significantly reducing MDA content in both hemolymph and hepatopancreas tissues. The predicted favorable MOS levels for immune- and antioxidant-related responses varied among tissues and indicators. Based on cubic regression analysis, selected parameters showed predicted peak values at intermediate MOS levels, but these estimates should be interpreted as model-based predictions within the tested range rather than definitive optimal doses. The observed enhancement in enzyme activity may be attributed to the potential of MOSs to interact with specific immune cells, thereby modulating or upregulating their enzymatic responses.

Previous studies have demonstrated that MOS supplementation enhances antioxidant and immune enzyme activities in various aquatic species. Chen et al. [42] reported that dietary MOSs increased the activities of T-AOC, AKP, and CAT in the serum of *Trachinotus ovatus*. Similarly, Lu et al. [9] observed significant improvements in T-AOC, MDA, and ACP activities in the serum of *E. sinensis* fed MOS-enriched diets. In milkfish (*Chanos chanos*), Harikrishnan et al. [21] found that MOS supplementation elevated SOD, CAT, and GSH activities. Moreover, Hunt et al. [43] showed that the inclusion of MOSs in Nile tilapia diets increased the activities of SOD, CAT, and GSH-Px. In *L. vannamei*, Chen et al. [44] reported improved CAT and SOD activities following dietary supplementation with a synbiotic containing MOSs and *Bacillus licheniformis*. Ren et al. [45] observed enhanced SOD, ACP, and T-AOC levels in hybrid grouper following MOS supplementation. Positive effects of MOSs on antioxidant enzyme systems have also been recorded in yellow catfish (*Pelteobagrus fulvidraco*) [46] and in grass carp fed diets containing prebiotic fibers, including MOSs and inulin (*C. idella*) [47]. Specifically, Lu [31] demonstrated that dietary MOSs significantly decreased MDA content in grass carp, suggesting reduced lipid peroxidation and enhanced oxidative stability.

Regarding the expression of immune-related functional genes, dietary MOS supplementation led to upregulation of GSH-Px, SOD, Trx, LZM, and ALF in the hepatopancreas of *C. quadricarinatus*. Among these, GSH-Px, SOD, and Trx are closely associated with antioxidant responses. The elevated expression levels of these genes indicate that MOSs enhance the antioxidant defense capacity of the crayfish. Meanwhile, LZM and ALF are linked to immune function. Their increased expression suggests that MOSs also contribute to the enhancement of the innate immune response in *C. quadricarinatus*.

The intestine serves as a vital organ for both digestion and immunity in crustaceans, with the intestinal microbiota playing a crucial role in regulating host growth and immune responses. In the present study, MOS supplementation exhibited no significant effects on alpha-diversity indices, including ACE, Chao1, Simpson, and Shannon indices, suggesting that microbial richness and diversity were not markedly altered. However, PCoA of beta diversity revealed that MOSs influenced the overall microbial community structure in the intestine of *C. quadricarinatus*. At the phylum level, Proteobacteria, Firmicutes, Bacteroidetes, and Fusobacteria were identified as the dominant phyla in the intestinal microbiota, consistent with previous findings [48]. Notably, MOSs increased the abundance of *Lactobacillus*, a genus of beneficial bacteria known to inhibit the proliferation of pathogenic microorganisms. This observation aligns with previous reports indicating that MOSs can promote the growth of beneficial microbes, including lactic acid bacteria, while suppressing pathogenic taxa [49]. Furthermore, the relative abundance of *Flavobacterium*, an opportunistic pathogen commonly implicated in aquatic animal diseases [50], was significantly reduced in MOS-treated groups, with the lowest abundance observed in the M3 group (0.6 g/kg). This suggests that MOSs may prevent the colonization of harmful bacteria in the gut, thereby contributing to intestinal homeostasis and improved host health.

*A. hydrophila* is a pathogenic bacterium responsible for a wide range of infectious diseases in aquatic animals, including septicemia, enteritis, ulcerative syndrome, and skin rot. These diseases are characterized by rapid onset, high infectivity, and elevated mortality rates. Due to the bacterial nature of these infections, antibiotics are commonly used for treatment. However, the widespread and often indiscriminate use of antibiotics in aquaculture has raised concerns regarding antibiotic residues and the emergence of antibiotic-resistant pathogenic bacteria, thereby complicating effective disease control [3]. Recent studies have demonstrated that MOS supplementation can enhance the resistance of various aquatic species to *A. hydrophila* infections. For instance, dietary MOSs have been shown to improve disease resistance in grass carp (*C. idella*) [51], blunt snout bream (*Megalobrama amblycephala*) [52], and *Labeo rohita* [53]. The findings of the present study are consistent with these reports, indicating that MOSs can significantly improve the resistance of *C. quadricarinatus* to *A. hydrophila* infection. These results provide a promising alternative strategy for mitigating the impact of *A. hydrophila* in aquaculture. Nevertheless, further investigations are required to elucidate the underlying molecular mechanisms of MOS-mediated disease resistance.

This study was conducted during the juvenile rearing stage under controlled tank conditions. Therefore, the long-term grow-out effects and economic cost–benefit balance of MOS supplementation under intensive commercial pond conditions require further evaluation. Future studies should also explore the molecular receptor pathways and mucosal immune mechanisms underlying the observed intestinal and immune responses.

## 5. Conclusions

In conclusion, dietary MOS supplementation improved growth performance, modulated the intestinal microbiota, enhanced immune- and antioxidant-related parameters, and increased resistance to *A. hydrophila* in juvenile *C. quadricarinatus*. Importantly, this study provides species-specific and integrated evidence in *C. quadricarinatus*, linking MOS-associated growth improvement with intestinal microbiota modulation, immune–antioxidant regulation, immune-related gene expression, and enhanced resistance to *A. hydrophila*. The favorable inclusion level varied among different endpoints, with growth performance peaking around 0.69 g/kg, ACP activity around 0.74 g/kg, and post-challenge survival being highest at 0.8 g/kg. Overall, intermediate MOS supplementation, particularly around 0.6–0.8 g/kg, appears to be beneficial for juvenile *C. quadricarinatus*.

## Figures and Tables

**Table 1:** Composition and nutrient level of experimental diets.

Ingredients	Content (%)
Soybean meal	21.0
Blood meal	2.0
Fish meal	13.0
Peanut meal	18.0
Flour	22.0
Corn starch	7.0
Cellulose	6.5
Monocalcium phosphate	1.5
Shell powder	1.0
Sodium alginate	2.0
Soybean oil	4.0
Vitamin premix ^a^	1.0
Mineral premix ^b^	1.0
Total	100
Nutrient levels (%)	
Crude protein	36.33
Crude lipid	5.81
Crude fiber	8.16
Moisture	6.35
Organic Matter	88.69
Ash	4.96
Gross energy (MJ/kg)	
16.92	

^a^ Vitamin premix provided by Hainan Zhengqiang Chaoyue Biochemical Technology Development Co., Ltd (Haikou, China). Vitamin premix included the following (mg kg^−1^ diet): vitamin A (9,000,000 IU), vitamin D3 (1,000,000 IU), vitamin E (18,000 IU), vitamin K3 (2000 mg), vitamin B1 (2000 mg), vitamin B2 (5000 mg), vitamin B6 (5000 mg), vitamin B12 (20 mg), vitamin C (8000 mg), calcium pantothenate (7000 mg), nicotinic acid (18,000 mg), folic acid (300 mg) and biotin (45 g). ^b^ Mineral premix included the following (mg kg^−1^ diet): CuSO_4_·5H_2_O (2 g), ZnSO_4_·H_2_O (25 g), MnSO_4_·H_2_O (15 g), FeSO_4_·H_2_O (25 g), KI (0.04 g), CoSO_4_·7H_2_O (0.1 g) and Na_2_SeO_3_ (0.02 g). Moisture content is reported on a wet-weight basis, whereas crude protein, crude lipid, and ash contents are expressed on a dry-matter basis.

**Table 2:** Primers used in quantitative real-time PCR.

Gene Name	Primer Sequence (5′-3′)	Amplification Efficiency (%)	Product Size (bp)
Beta-actin (β-actin)	F: ATCACTGCTCTGGCTCCTGCTACC	108	148
	R: CGGACTCGTCGTACTCCTCCTTGG		
Glutathione peroxidase (GSH-Px)	F: CAAGGTGGTGTTGGTGGTCA	103	228
	R: TCAAAGAAGGTCATCAGAGGCT		
Superoxide dismutase (SOD)	F: CAAAATTCCCTTGCCAGCAC	100	124
	R: CCTGGTTCCAGCTTGATGTT		
Thioredoxin (Trx)	F: CATTAGCGCCAGAGAAGGAC	104	142
	R: ATGTAGCCATGGAACACCAG		
Lysozyme (LZM)	F: GCAACAGGAACGGTAGCAAG	107	190
	R: GCCACCCAAGCGGAATAT		
Anti-lipopolysaccharide factor (ALF)	F: ACAGAGCAACGCACAGATTACA	105	176
	R: TCCAGCCAGGGCAATACAT		

**Table 3:** Effects of MOSs on growth performance of *C. quadricarinatus*.

	M0	M1	M2	M3	M4	M5
IW (g)	0.29 ± 0.01	0.29 ± 0.00	0.28 ± 0.01	0.27 ± 0.01	0.27 ± 0.02	0.28 ± 0.01
FW (g)	3.64 ± 0.07 ^b^	3.94 ± 0.07 ^ab^	3.89 ± 0.19 ^ab^	4.16 ± 0.15 ^a^	4.00 ± 0.13 ^ab^	3.93 ± 0.22 ^ab^
WGR (%)	1152.81 ± 36.04 ^b^	1272.43 ± 44.02 ^ab^	1296.73 ± 75.14 ^ab^	1426.30 ± 138.91 ^a^	1367.78 ± 145.50 ^ab^	1304.46 ± 83.39 ^ab^
SGR (%/d)	4.51 ± 0.05 ^b^	4.68 ± 0.06 ^ab^	4.71 ± 0.10 ^ab^	4.86 ± 0.16 ^a^	4.79 ± 0.18 ^ab^	4.72 ± 0.11 ^ab^
SR (%)	75.83 ± 1.44 ^a^	78.33 ± 5.77 ^a^	84.17 ± 3.82 ^a^	80.83 ± 2.89 ^a^	80.00 ± 2.50 ^a^	78.33 ± 6.29 ^a^
FCR	1.47 ± 0.10 ^a^	1.41 ± 0.25 ^a^	1.78 ± 0.05 ^a^	1.48 ± 0.10 ^a^	1.77 ± 0.07 ^a^	1.70 ± 0.34 ^a^

Values are presented as means ± SDs based on three replicate tanks per treatment. Different superscript letters within the same row indicate significant differences among treatments (*p* < 0.05), whereas values sharing the same letter are not significantly different.

**Table 4:** Effects of MOSs on muscle composition of *C. quadricarinatus*.

	M0	M1	M2	M3	M4	M5
Moisture (%)	77.78 ± 0.73 ^a^	78.37 ± 0.61 ^a^	77.79 ± 0.35 ^a^	78.49 ± 0.43 ^a^	76.09 ± 2.28 ^a^	78.30 ± 0.44 ^a^
Crude protein (%)	84.43 ± 0.52 ^a^	84.73 ± 3.37 ^a^	84.66 ± 0.61 ^a^	86.56 ± 4.00 ^a^	84.53 ± 2.97 ^a^	85.72 ± 2.85 ^a^
Crude lipid (%)	3.84 ± 0.05 ^a^	3.41 ± 0.06 ^b^	3.39 ± 0.08 ^b^	3.26 ± 0.08 ^b^	3.29 ± 0.01 ^b^	3.29 ± 0.08 ^b^
Ash (%)	6.59 ± 0.31 ^a^	6.21 ± 0.26 ^a^	6.15 ± 0.16 ^a^	6.12 ± 0.22 ^a^	6.20 ± 0.37 ^a^	6.51 ± 0.31 ^a^

Values are presented as means ± SD. Moisture content is reported on a wet-weight basis, whereas crude protein, crude lipid, and ash contents are expressed on a dry-matter basis. Different superscript letters within the same row indicate significant differences among treatments (*p* < 0.05).

**Table 5:** Effects of MOSs on hemolymph immune-related physiological and biochemical indices of *C. quadricarinatus*.

	M0	M1	M2	M3	M4	M5
AKP (U/100 mL)	22.83 ± 1.52 ^c^	34.64 ± 0.58 ^b^	35.45 ± 1.22 ^b^	42.66 ± 1.46 ^a^	33.75 ± 1.00 ^b^	33.52 ± 1.15 ^b^
ACP (U/100 mL)	7.30 ± 0.48 ^c^	9.92 ± 0.23 ^b^	10.24 ± 0.63 ^b^	11.39 ± 0.32 ^a^	11.21 ± 0.28 ^a^	11.16 ± 0.18 ^a^
T-AOC (mmol/L)	0.87 ± 0.06 ^d^	1.00 ± 0.07 ^bc^	1.09 ± 0.05 ^b^	1.11 ± 0.05 ^a^	0.91 ± 0.08 ^cd^	0.92 ± 0.02 ^cd^
SOD (U/mL)	101.41 ± 6.18 ^c^	119.91 ± 2.71 ^b^	127.41 ± 4.15 ^a^	128.59 ± 3.17 ^a^	124.01 ± 4.96 ^ab^	105.55 ± 2.50 ^c^
CAT (U/mL)	3.52 ± 0.28 ^a^	3.79 ± 0.23 ^a^	3.87 ± 0.19 ^a^	3.93 ± 0.34 ^a^	3.70 ± 0.16 ^a^	3.65 ± 0.27 ^a^
GSH-Px (U/mL)	267.50 ± 9.75 ^c^	378.33 ± 6.45 ^b^	412.08 ± 6.97 ^a^	417.08 ± 6.60 ^a^	411.67 ± 8.32 ^a^	407.50 ± 10.48 ^a^
MDA (nmol/mL)	3.94 ± 0.32 ^a^	2.69 ± 0.23 ^b^	1.88 ± 0.24 ^de^	1.77 ± 0.23 ^e^	2.23 ± 0.12 ^cd^	2.33 ± 0.25 ^bc^

Values are presented as means ± SDs. Different superscript letters within the same row indicate significant differences among treatments (*p* < 0.05), whereas values sharing the same letter are not significantly different.

**Table 6:** Effects of MOSs on physiological and biochemical indices related to hepatopancreas of *C. quadricarinatus*.

	M0	M1	M2	M3	M4	M5
AKP (U/g prot)	28.92 ± 2.22 ^d^	31.46 ± 1.00 ^cd^	34.78 ± 1.26 ^b^	39.86 ± 1.86 ^a^	33.50 ± 1.40 ^bc^	30.92 ± 0.66 ^cd^
ACP (U/g prot)	194.00 ± 5.01 ^c^	201.95 ± 5.81 ^b^	210.14 ± 2.48 ^a^	213.24 ± 4.61 ^a^	213.91 ± 2.34 ^a^	210.94 ± 4.03 ^a^
T-AOC (mmol/g prot)	0.13 ± 0.01 ^b^	0.16 ± 0.01 ^a^	0.16 ± 0.01 ^a^	0.17 ± 0.01 ^a^	0.17 ± 0.00 ^a^	0.16 ± 0.01 ^a^
SOD (U/mg prot)	21.93 ± 1.30 ^c^	24.38 ± 1.59 ^bc^	29.63 ± 3.05 ^a^	25.83 ± 1.54 ^b^	22.45 ± 1.54 ^bc^	22.08 ± 2.11 ^c^
CAT (U/mg prot)	7.76 ± 1.04 ^c^	9.33 ± 0.65 ^c^	15.20 ± 1.37 ^a^	15.48 ± 0.67 ^a^	11.63 ± 1.46 ^b^	11.74 ± 1.17 ^b^
GSH-Px (U/mg prot)	31.17 ± 3.64 ^b^	37.71 ± 4.18 ^a^	39.92 ± 3.49 ^a^	38.42 ± 3.17 ^a^	40.20 ± 1.50 ^a^	39.72 ± 2.59 ^a^
MDA (nmol/mg prot)	2.21 ± 0.14 ^a^	1.62 ± 0.13 ^c^	1.26 ± 0.14 ^d^	1.65 ± 0.12 ^bc^	1.71 ± 0.13 ^bc^	1.86 ± 0.09 ^b^

Values are presented as means ± SDs. Different superscript letters within the same row indicate significant differences among treatments (*p* < 0.05), whereas values sharing the same letter are not significantly different.

**Table 7:** Alpha-diversity index statistics of intestinal microorganisms of *C. quadricarinatus*.

	M0	M1	M2	M3	M4	M5
Chao1	598.47 ± 107.78 ^a^	642.23 ± 11.64 ^a^	571.00 ± 29.44 ^a^	557.33 ± 92.81 ^a^	560.67 ± 71.00 ^a^	629.02 ± 67.90 ^a^
Simpson	0.95 ± 0.00 ^a^	0.97 ± 0.01 ^a^	0.96 ± 0.01 ^a^	0.95 ± 0.03 ^a^	0.95 ± 0.02 ^a^	0.97 ± 0.02 ^a^
Shannon	5.84 ± 0.15 ^a^	6.67 ± 0.63 ^a^	6.07 ± 0.38 ^a^	5.72 ± 1.04 ^a^	5.85 ± 0.55 ^a^	7.12 ± 1.68 ^a^

Values are presented as means ± SDs (n = 3 biological replicates per treatment). Differences among groups were analyzed using the Kruskal–Wallis test. No significant differences were observed among treatments (*p* > 0.05). Values in the same row with the same lowercase letter are not significantly different (*p* > 0.05), based on one-way ANOVA and Duncan’s multiple-range test.

## Data Availability

The original contributions presented in this study are included in the article/Supplementary Material. Further inquiries can be directed to the corresponding authors.

## Supplementary Materials

The following supporting information can be downloaded at https://www.mdpi.com/article/10.3390/ani16162511/s1, Table S1: Basic information on high-pass sequencing of intestinal microorganisms of *C. quadricarinatus*.

## References

[1] Asche F., Cojocaru A.L., Roth B. (2018). The development of large scale aquaculture production: A comparison of the supply chains for chicken and salmon. Aquaculture.

[2] Alfiansah Y.R., Hassenrück C., Kunzmann A., Taslihan A., Harder J., Gärdes A. (2018). Bacterial abundance and community composition in pond water from shrimp aquaculture systems with different stocking densities. Front Microbiol..

[3] Pepi M., Focardi S. (2021). Antibiotic-resistant bacteria in aquaculture and climate change: A challenge for health in the Mediterranean area. Int. J. Environ. Res. Public Health.

[4] Wang W., Sun J., Liu C., Xue Z. (2017). Application of immunostimulants in aquaculture: Current knowledge and future perspectives. Aquac. Res..

[5] Shi F., Lu Z., Yang M., Li F., Zhan F., Zhao L., Li Y., Li Q., Li J., Li J. (2021). Astragalus polysaccharides mediate the immune response and intestinal microbiota in grass carp (*Ctenopharyngodon idellus*). Aquaculture.

[6] Faustino M., Durão J., Pereira C.F., Pintado M.E., Carvalho A.P. (2021). Mannans and mannan oligosaccharides (MOS) from *Saccharomyces cerevisiae*–A sustainable source of functional ingredients. Carbohyd Polym..

[7] Kamal M., Zhu L., Abd El-Hack M.E., Arif M., Li F., Cheng Y. (2025). Functional Roles of Mannan and Chitosan Oligosaccharides on Animal Health and Nutrition: A Review. Carbohydr. Polym. Technol. Appl..

[8] Mugwanya M., Dawood M.A., Kimera F., Sewilam H. (2022). Updating the role of probiotics, prebiotics, and synbiotics for tilapia aquaculture as leading candidates for food sustainability: A review. Probiotics Antimicrob. Proteins.

[9] Lu J., Qi C., Limbu S.M., Han F., Yang L., Wang X., Qin J.G., Chen L. (2019). Dietary mannan oligosaccharide (MOS) improves growth performance, antioxidant capacity, non-specific immunity and intestinal histology of juvenile Chinese mitten crabs (*Eriocheir sinensis*). Aquaculture.

[10] Torrecillas S., Makol A., Benítez-Santana T., Caballero M.J., Montero D., Sweetman J., Izquierdo M. (2011). Reduced gut bacterial translocation in European sea bass (*Dicentrarchus labrax*) fed mannan oligosaccharides (MOS). Fish. Shellfish Immun..

[11] Oyofo B.A., DeLoach J.R., Corrier D.E., Norman J.O., Ziprin R.L., Mollenhauer H.H. (1989). Prevention of *Salmonella typhimurium* colonization of broilers with D-mannose. Poult. Sci..

[12] Ahyong S.T., Yeo D.C. (2007). Feral populations of the Australian red-claw crayfish (*Cherax quadricarinatus* von Martens) in water supply catchments of Singapore. Biol. Invasions.

[13] Saoud I.P., Ghanawi J., Thompson K.R., Webster C.D. (2013). A review of the culture and diseases of redclaw crayfish *Cherax quadricarinatus* (von Martens 1868). J. World Aquacult. Soc..

[14] Greco L.S.L., Stumpf L., Timpanaro S., Cid A.R., Lamberti M., Battista A., LauraTomas A., Jones C.M. (2022). Impact of low-cost diets on maturation of the red claw crayfish *Cherax quadricarinatus*: An integrative approach during a long-term study. Aquaculture.

[15] Jones C.M. (1995). Production of juvenile redclaw crayfish, *Cherax quadricarinatus* (von Martens)(Decapoda, Parastacidae) I. Development of hatchery and nursery procedures. Aquaculture.

[16] Naranjo-Páramo J., Hernández-Llamas A., Vargas-Mendieta M., Villarreal-Colmenares H. (2021). Stochastic dynamic model analysis of the effect of stocking density on the monosex production of male redclaw crayfish *Cherax quadricarinatus* reared in commercial gravel-lined ponds. Aquaculture.

[17] Wu D., Huang Y., Chen Q., Jiang Q., Li Y., Zhao Y. (2019). Effects and transcriptional responses in the hepatopancreas of red claw crayfish *Cherax quadricarinatus* under cold stress. J. Therm. Biol..

[18] Wang T., Yang J., Lin G., Li M., Zhu R., Zhang Y., Mai K. (2021). Effects of dietary mannan oligosaccharides on non-specific immunity, intestinal health, and antibiotic resistance genes in pacific white shrimp *Litopenaeus vannamei*. Front. Immunol..

[19] Novriadi R., Fayuan G., Davies S., Istiqomah I., Isnansetyo A., Farkan M., Jinjun D., Jianhua Y., Xin H., Yan Z. (2024). Evaluation of dietary yeast derived mannan oligosaccharide for pacific whiteleg shrimp *Penaeus vannamei*: Effects on growth performance, immune response, hepatopancreas morphology and resilience to infection against *Vibrio parahaemolyticus*. Aquacult. Rep..

[20] Hoang D., Ky P.X., Thi V.H. (2023). Dietary mannan oligosaccharides elevated growth performance, gut morphology, microbiota, body composition, feed and nutrient utilisation of pompano, *Trachinotus ovatus*. Aquacult. Rep..

[21] Harikrishnan R., Devi G., Balamurugan P., Abdel-Warith A.A., Younis E.M., Van Doan H., Balasundaram C., Davies S.J., El-Haroun E. (2023). Immunostimulatory effect of mannan-oligosaccharides supplementation diet in milkfish (*Chanos chanos*). Fish. Shellfish Immun..

[22] Liu J., Li E., Li X., Wang X., Huang Q., Wang H., Miao Y., Shi Q., Qin J., Chen L. (2024). Effects of dietary methionine on the growth and protein synthesis of juvenile Chinese mitten crabs (*Eriocheir sinensis*) fed fish meal-free diets. Anim. Nutr..

[23] Zhang X., Cheng K., Dong L., Zhu D., Li Q., Guo Z., Luo Y., Liu A., Tian J., Peng D. (2025). Mechanisms of hepatic dysfunction in Nile Tilapia (*Oreochromis Niloticus*) fed a figh-fava bean (*Vicia faba* L.) diet. Anim. Nutr..

[24] Wang M., Feng L., Wu P., Liu Y., Ren H., Jin X., Zhou X., Jiang W. (2025). Multiple ways to promote adult grass carp (*Ctenopharyngodon idella*) muscle hypertrophy: Application of dietary myo-inositol. Anim. Nutr..

[25] Yuan H., Hu J., Li X., Sun Q., Tan X., You C., Dong Y., Huang Y., Zhou M. (2024). Dietary black soldier fly oil enhances growth performance, flesh quality, and health status of largemouth bass (*Micropterus salmoides*). Anim. Nutr..

[26] Huang C., Li P., Ma X., Jaworski N.W., Stein H., Lai C., Zhao J., Zhang S. (2018). Methodology effects on determining the energy concentration and the apparent total tract digestibility of components in diets fed to growing pigs. Asian Austral. J. Anim..

[27] Lu Y.P., Zheng P.H., Xu J.R., Cao Y.L., Li J.T., Hao C.G., Zhang Z.L., Xian J.A., Zhang X.X., Wang A.L. (2023). Effects of dietary *Eucommia ulmoides* leaf extract on growth, muscle composition, hepatopancreas histology, immune responses and microcystin-LR resistance of juvenile red claw crayfish (*Cherax quadricarinatus*). Fishes.

[28] Wang T., Wu H., Li W., Xu R., Qiao F., Du Z., Zhang M. (2022). Effects of dietary mannan oligosaccharides (MOS) supplementation on metabolism, inflammatory response and gut microbiota of juvenile Nile tilapia (*Oreochromis niloticus*) fed with high carbohydrate diet. Fish. Shellfish Immun..

[29] Zhang J., Liu Y., Tian L., Yang H., Liang G., Xu D. (2012). Effects of dietary mannan oligosaccharide on growth performance, gut morphology and stress tolerance of juvenile Pacific white shrimp, *Litopenaeus vannamei*. Fish. Shellfish Immun..

[30] Akter M.N., Zahan K., Zafar M.A., Khatun N., Rana M.S., Mursalin M.I. (2021). Effects of dietary mannan oligosaccharide on growth performance, feed utilization, body composition and haematological parameters in Asian catfish (*Clarias batrachus*) juveniles. Turk. J. Fish. Aquat. SC..

[31] Lu Z., Feng L., Jiang W., Wu P., Liu Y., Kuang S., Tang L., Zhou X. (2020). Mannan oligosaccharides improved growth performance and antioxidant capacity in the intestine of on-growing grass carp (*Ctenopharyngodon idella*). Aquacult. Rep..

[32] Gelibolu S., Yanar Y., Genc M.A., Genc E. (2018). The effect of mannan-oligosaccharide (MOS) as a feed supplement on growth and some blood parameters of Gilthead Sea Bream (*Sparus aurata*). Turk. J. Fish. Aquat. Sc..

[33] Xu B., Wu S., Han Q. (2021). Modulation of the growth performance and innate immunity of loaches (*Paramisgurnus dabryanus*) upon dietary mannan oligosaccharides. 3 Biotech.

[34] Dimitroglou A., Merrifield D.L., Moate R., Davies S.J., Spring P., Sweetman J., Bradley G. (2009). Dietary mannan oligosaccharide supplementation modulates intestinal microbial ecology and improves gut morphology of rainbow trout, *Oncorhynchus mykiss* (Walbaum). J. Anim. Sci..

[35] Do-Huu H., Thuy N.T.T., Ky P.X. (2024). The ameliorative roles of dietary mannan oligosaccharides and vitamin E on growth performance, intestinal microbes, and structure, flesh quality, nutrient efficacy and stress resistance of Pompano, *Trachinotus ovatus*. Aquacult. Rep..

[36] Dimitroglou A., Merrifield D.L., Spring P., Sweetman J., Moate R., Davies S.J. (2010). Effects of mannan oligosaccharide (MOS) supplementation on growth performance, feed utilisation, intestinal histology and gut microbiota of gilthead sea bream (*Sparus aurata*). Aquaculture.

[37] Mohammadian T., Nasirpour M., Tabandeh M.R., Mesbah M. (2019). Synbiotic effects of β-glucan, mannan oligosaccharide and *Lactobacillus casei* on growth performance, intestine enzymes activities, immune-hematological parameters and immune-related gene expression in common carp, *Cyprinus carpio*: An experimental infection with *Aeromonas hydrophila*. Aquaculture.

[38] Yoon D.S., Kim D.H., Kim J.H., Sakakura Y., Hagiwara A., Park H.G., Lee M.C., Lee J.S. (2024). Interactions between lipid metabolism and the microbiome in aquatic organisms: A review. Mar. Pollut. Bull..

[39] Piccolo G., Centoducati G., Bovera F., Marrone R., Nizza A. (2013). Effects of mannan oligosaccharide and inulin on sharpsnout seabream (*Diplodus puntazzo*) in the context of partial fish meal substitution by soybean meal. Ital. J. Anim. Sci..

[40] Du J., Zhu H., Liu P., Chen J., Xiu Y., Yao W., Wu T., Ren Q., Meng Q., Gu W. (2013). Immune responses and gene expression in hepatopancreas from *Macrobrachium rosenbergii* challenged by a novel pathogen spiroplasma MR-1008. Fish. Shellfish Immun..

[41] Zhang Y., Ye C., Wang A., Zhu X., Chen C., Xian J., Sun Z. (2015). Isolated and combined exposure to ammonia and nitrite in giant freshwater pawn *(Macrobrachium rosenbergii*): Effects on the oxidative stress, antioxidant enzymatic activities and apoptosis in haemocytes. Ecotoxicology.

[42] Chen Z., Wu Y., Cai Y., Chen X., Zhou Y., Cao Z., Li J., Wang S. (2024). Mannan oligosaccharide improves antioxidant capacity, non-specific immunity and protection against Vibrio disease and Typhoon stress in. *Trachinotus ovatus* juveniles. Isr. J. Aquac.-Bamidgeh.

[43] Özlüer-Hunt A., Berköz M., Özkan F., Yalin S., Erçen Z., Erdoğan E., Gündüz S.G. (2011). Effect of mannan oligosaccharide on growth, body composition, and antioxidant enzyme activity of tilapia (*Oreochromis niloticus*). Isr. J. Aquac.-Bamidgeh IIC.

[44] Chen M., Chen X., Tian L., Liu Y., Niu J. (2020). Beneficial impacts on growth, intestinal health, immune responses and ammonia resistance of pacific white shrimp (*Litopenaeus vannamei*) fed dietary synbiotic (mannan oligosaccharide and *Bacillus licheniformis*). Aquacult. Rep..

[45] Ren Z., Wang S., Cai Y., Wu Y., Tian L., Wang S., Jiang L., Guo W., Sun Y., Zhou Y. (2020). Effects of dietary mannan oligosaccharide supplementation on growth performance, antioxidant capacity, non-specific immunity and immune-related gene expression of juvenile hybrid grouper (*Epinephelus lanceolatus*♂ × *Epinephelus fuscoguttatus*♀). Aquaculture.

[46] Wu Z., Yu Y., Chen X., Liu H., Yuan J., Shi Y., Chen X. (2014). Effect of prebiotic konjac mannanoligosaccharide on growth performances, intestinal microflora, and digestive enzyme activities in yellow catfish, *Pelteobagrus fulvidraco*. Fish. Physiol. Biochem..

[47] Mo W.Y., Cheng Z., Choi W.M., Lun C.H., Man Y.B., Wong J.T., Chen X.W., Lau S.C., Wong M.H. (2015). Use of food waste as fish feeds: Effects of prebiotic fibers (inulin and mannanoligosaccharide) on growth and non-specific immunity of grass carp (*Ctenopharyngodon idella*). Environ. Sci. Pollut. Res..

[48] Zhang Z., Cao Y., Xu J., Zhang X., Li J., Li J., Zheng P., Xian J., Lu Y. (2024). Effects of dietary chitosan oligosaccharide on the growth, intestinal microbiota and immunity of juvenile red claw crayfish (*Cherax quadricarinatus*). Fish. Shellfish Immun..

[49] Akter M.N., Sutriana A., Talpur A.D., Hashim R. (2016). Dietary supplementation with mannan oligosaccharide influences growth, digestive enzymes, gut morphology, and microbiota in juvenile striped catfish, *Pangasianodon hypophthalmus*. Aquacult. Int..

[50] Pękala-Safińska A. (2018). Contemporary threats of bacterial infections in freshwater fish. J. Vet. Res..

[51] Lu Z., Feng L., Jiang W., Wu P., Liu Y., Jiang J., Kuang S., Tang L., Li S., Zhong C. (2022). Dietary mannan oligosaccharides strengthens intestinal immune barrier function via multipath cooperation during *aeromonas hydrophila* infection in grass carp (*Ctenopharyngodon idella*). Front Immunol..

[52] Ding Z., Wang X., Liu Y., Zheng Y., Li H., Zhang M., He Y., Cheng H., Xu J., Chen X. (2022). Dietary mannan oligosaccharides enhance the non-specific immunity, intestinal health, and resistance capacity of juvenile blunt snout bream (*Megalobrama amblycephala*) against *aeromonas hydrophila*. Front Immunol..

[53] Andrews S.R., Sahu N.P., Pal A.K., Kumar S. (2009). Haematological modulation and growth of *Labeo rohita* fingerlings: Effect of dietary mannan oligosaccharide, yeast extract, protein hydrolysate and chlorella. Aquac. Res..

