# Supplementary material for: Modulation of Growth, Intestinal Microbiota, and Immune Responses in Cherax quadricarinatus by Mannan Oligosaccharides
Source: Animals (Basel). 2026 Aug 12;16(16):2511. doi: 10.3390/ani16162511 (PMC13508865; doi:10.3390/ani16162511)
Supplement: Supplementary file 1 [file animals-16-02511-s001.zip › animals-4387765-supplementary.pdf]

Table S1 Basic information of high-pass sequencing of intestinal microorganisms of *C. quadricarinatus*

| Sample ID | Raw Reads | Clean Reads | Effective Reads | OTU |
|---|---|---|---|---|
| M0a | 160031 | 159638 | 145126 | 578 |
| M0b | 159997 | 159627 | 148454 | 715 |
| M0c | 159790 | 159377 | 147994 | 502 |
| M1a | 159744 | 159359 | 147515 | 634 |
| M1b | 147391 | 147072 | 133171 | 1105 |
| M1c | 159940 | 159535 | 148442 | 650 |
| M2a | 159942 | 159558 | 148380 | 588 |
| M2b | 160184 | 159783 | 146453 | 588 |
| M2c | 159723 | 159338 | 146907 | 537 |
| M3a | 160085 | 159722 | 144516 | 520 |
| M3b | 159790 | 159413 | 131189 | 663 |
| M3c | 160049 | 159683 | 141960 | 489 |
| M4a | 105798 | 105576 | 92171 | 482 |
| M4b | 133856 | 133537 | 122676 | 580 |
| M4c | 159755 | 159413 | 145167 | 620 |
| M5a | 160199 | 159803 | 144341 | 677 |
| M5b | 160000 | 159588 | 143412 | 581 |
| M5c | 160290 | 159892 | 138893 | 2879 |
